# Current Status and Perspectives on Wire and Arc Additive Manufacturing (WAAM)

**DOI:** 10.3390/ma12071121

**Published:** 2019-04-04

**Authors:** Tiago A. Rodrigues, V. Duarte, R. M. Miranda, Telmo G. Santos, J. P. Oliveira

**Affiliations:** UNIDEMI, Departamento de Engenharia Mecânica e Industrial, Faculdade de Ciências e Tecnologia, Universidade NOVA de Lisboa, 2829-516 Caparica, Portugal; v.duarte@campus.fct.unl.pt (V.D.); rmmdm@fct.unl.pt (R.M.M.); telmo.santos@fct.unl.pt (T.G.S.)

**Keywords:** wire and arc additive manufacturing, additive manufacturing, microstructure, mechanical properties, applications

## Abstract

Additive manufacturing has revolutionized the manufacturing paradigm in recent years due to the possibility of creating complex shaped three-dimensional parts which can be difficult or impossible to obtain by conventional manufacturing processes. Among the different additive manufacturing techniques, wire and arc additive manufacturing (WAAM) is suitable to produce large metallic parts owing to the high deposition rates achieved, which are significantly larger than powder-bed techniques, for example. The interest in WAAM is steadily increasing, and consequently, significant research efforts are underway. This review paper aims to provide an overview of the most significant achievements in WAAM, highlighting process developments and variants to control the microstructure, mechanical properties, and defect generation in the as-built parts; the most relevant engineering materials used; the main deposition strategies adopted to minimize residual stresses and the effect of post-processing heat treatments to improve the mechanical properties of the parts. An important aspect that still hinders this technology is certification and nondestructive testing of the parts, and this is discussed. Finally, a general perspective of future advancements is presented.

## 1. Introduction

Additive manufacturing is nowadays one of the hot topics in the manufacturing and engineering worlds. The ability to create three-dimensional, complex, and near-net shape parts in a layer by layer deposition process is currently a major driving force for major breakthroughs. These breakthroughs are observed either in the process itself, by developing process variants with dedicated purposes to increase capabilities, but also on the materials used, since the non-equilibrium solidification conditions which occur during fusion-based additive manufacturing can lead to microstructural features not often found in conventional materials manufacturing processes. 

Currently, additive manufacturing processes based on fusion are mostly focused on powder-bed systems using laser and/or electron beams as heat sources. Despite very high precision dimensional tolerances achieved with these techniques, the deposition rate is low, increasing lead times. Additionally, using powder as the feedstock materials makes the process more prone to the formation of defects such as pores, which can hamper the structural integrity of the parts, especially during dynamic solicitation conditions. Wire and arc additive manufacturing (WAAM) uses an electric arc as the heat source and a solid wire as the feedstock material. Though the precision of the as-built parts may be lower than those obtained using powder-bed systems, the depositions rates are significantly higher, allowing to manufacture large metallic structural parts in short times. WAAM is currently being embraced by both academia and industry owing to the advantages of the technique. Therefore, several research papers have been dealing with fundamental aspects associated with the process and its effects on the material’s microstructure and mechanical properties. Additionally, some applications are already making use of parts built by WAAM, showing the viability of this process. This review paper intends to provide an overview of the major developments in WAAM, covering important topics, such as process variants, materials for WAAM, development of residual stresses, and post-processing heat treatment, as well as non-destructive testing. This review finalizes with current applications based on WAAM parts and a summary of areas where additional research efforts need to focus.

## 2. Wire and Arc Additive Manufacturing (WAAM) Process and Variants

**Figure 1:**
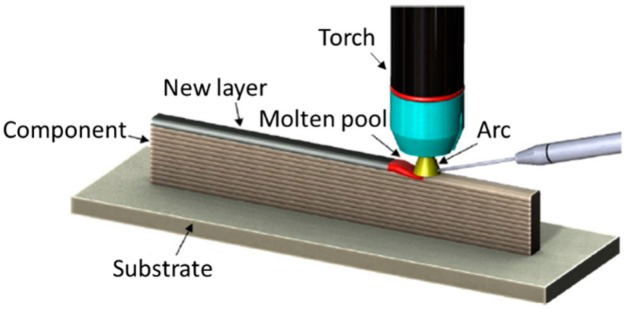
Schematic representation of the wire and arc additive manufacturing (WAAM) process (adapted from [6]).

WAAM is classified in the category of direct energy deposition according to ASTM F2792-12a [1], and is defined as the combination of an electric arc used as a heat source, and a wire employed as a feedstock material. The process is schematically represented in Figure 1. WAAM relies on the fundamental concepts of automatized welding processes, such as: gas metal arc welding (GMAW) [2], plasma arc welding (PAW) [3], and gas tungsten arc welding (GTAW) [4]. 

In the past years, WAAM has had different designations, such as rapid prototyping (RP), shape welding (SW), shape melting (SM), solid freeform fabrication (SFF), shape metal deposition (SMD), and even 3D welding [5].

From the existing arc welding processes, GMAW, also known as metal inert gas (MIG)/metal active gas (MAG), is the most used process in WAAM. GMAW is a fusion-based arc welding process where the arc is established between the tip of a consumable wire and the workpiece under the protection of an inert or active shielding gas that also protects the weld pool and adjacent material. Deposition rates range from 15 to 160 g/min using GMAW in additive manufacturing, depending on the deposited material and process parameters, making it ideal for the production of large-scale parts in short time spans [7,8,9].

The other two arc welding processes are GTAW and PAW. These have some similarities, since they both use a non-consumable tungsten electrode to establish an electric arc with the workpiece under an inert shielding gas without filler material. GTAW was one of the first arc welding processes that became widespread due to its high precision with almost no defects, since the electric arc is very stable [10]. However, for WAAM it needs external filler material. PAW is a high energy-density process, where the arc is forced to pass through an orifice placed between the cathode and anode that constrains the arc, resulting in an increased arc stability. Through the use of a mostly inert, plasmogenic arc, a highly localized ionized plasma forms with very high temperatures and energy. Thus, it is considered a high-density energy process with energy densities lower than those obtained by high power beams as a laser [11], but higher than other electric arc processes. The heat affected zone (HAZ) of PAW beads is narrow and thin, allowing for better control of weld bead geometry due to the increased flexibility for independently controlling the most important process parameters: current and wire feed speed [12]. By varying the plasma gas flow rate, torch orifice diameter, and current intensity, it is possible to achieve different operating modes in PAW namely microplasma, medium current, and keyhole plasma. Microplasma is characterized by requiring low welding currents, between 0.1 and 15 A. This welding technique, when applied to additive manufacturing, presents major advantages regarding the total wall width (TWW) by allowing the production of thin parts with TWW values as low as 2 mm. Though deposition rates are low, about 1.0 g/min [13], when compared to other arc based processes, the low heat input allows for good surface finish. The most commonly used operation mode in PAW is medium current, with the current typically ranging from 15 to 200 A. This operation mode has very similar characteristics to GTAW, but the plasma confinement makes the arc stiffer and less sensitive to torch stand-off variations. Wall width varies from 4 to 15 mm and the deposition rate can be as high as 30 g/min [3,7,14]. Keyhole mode is characterized by high penetration, making it unsuitable for additive manufacturing, since it fully melts the previously deposited layers compromising the wall stability and geometric accuracy.

Electric arc-based welding, in general, can be complex since several process parameters must be controlled to have a good quality of the final parts. Process parameters include: current intensity, voltage, shielding gas type and flow rate, contact-tip-to-work distance, wire feed speed, travel speed, and torch angle. Thus, for each equipment and materials involved, these have to be optimized. The right selection of parameters affects the transfer mode, which is very important to determine bead width, penetration and size, deposition rate, and surface roughness. 

In the pursuit for a better and more stable process to control molten metal deposition with reduced heat input, a variant of GMAW, known as cold metal transfer (CMT), has been adapted to additive manufacturing. It is an advanced material transfer process in which an incorporated control system detects when the electrode wire tip contacts with the molten pool, and by activation of a servomotor, retracts the wire in a push and pull electromechanical process, to control droplet transfer. Variants of cold metal transfer include CMT pulse (CMT-P), CMT advanced (CMT-ADV), and CMT pulse advanced (CMT-PADV), which have been developed by Fronius [15]. When optimized, cold metal transfer is suitable for application in Ti-based alloys [16]. 

Another variant is tandem GMAW, in which two wires are fed into the melt pool in order to achieve high deposition rates (160 g/min) [9,17]. Nevertheless, this method requires a high amount of energy to maintain the arc, so some improvements that enhance heat dissipation are required to control the molten pool shape.

Arc welding-based technologies have been successfully used for additive manufacturing applications, especially because there is considerable knowledge accumulated on process, welding metallurgy, and mechanical performance of welded parts. However, in WAAM there are issues which still need research, such as determination of optimum torch path planning to obtain fully dense parts with minimized residual stresses, control of microstructural evolution during multiple layer deposition, and effect of temperature between layers, among others. Some research groups have developed new process variants to mitigate some of the above-mentioned issues, namely by applying mechanical deformation between layers or active heating and cooling, for example.

### 2.1. WAAM Process Variants

#### 2.1.1. Cold-Work Based Techniques 

**Figure 2:**
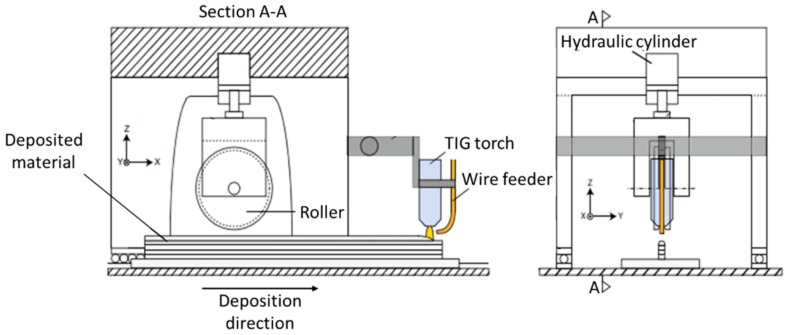
Schematic diagram of cold rolling WAAM process (adapted from [18]).

High pressure inter-layer rolling is a cold-work process variant developed at Cranfield University [18]. It consists of imposing a load of up to 100 kN onto a roller traveling over the already existent deposited layers, to promote plastic deformation of the surface and thus recrystallize the grain in the following deposition. The process is schematically depicted in Figure 2. 

Martina et al. [18] showed that inter-layer rolling in WAAM of Ti-6Al-4V induced prior β grain refinement, thickness reduction of the α-phase lamellae, and an overall modification of the microstructure from columnar to equiaxed. After these promising results, several studies have been conducted to extract other benefits from the high pressure inter-layer rolling, particularly to control residual stress and distortion in aluminum [19,20] and titanium [21,22] alloys. In order to strengthen the as-deposited WAAM material, Gu et al. [14,23] combined inter-layer rolling with a post-WAAM heat treatment, achieving higher mechanical properties (ultimate tensile strength and elongation) with the increase of the applied load. 

**Figure 3:**
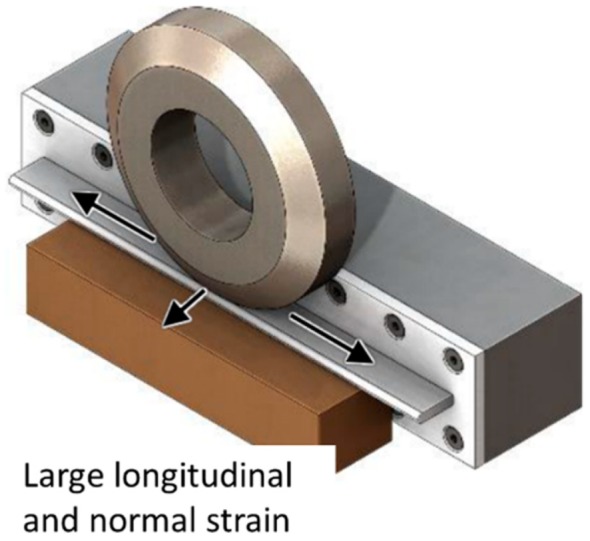
Schematic representation of side rolling of WAAM part to enhance surface finish (adapted from [19]).

Porosity is a recurrent problem in WAAM, particularly in aluminum alloys, due to its low hydrogen solubility in the solid-sate having a preferential location at the layer boundaries, which causes a decrease of mechanical properties when stressed in the perpendicular direction. Inter-layer rolling decreases porosity size and quantity with increasing rolling load, which has been claimed as the reason for the ductility increase of WAAM-rolled aluminum alloys [24]. Roller design plays an important role in this methodology and its geometry must be adapted according to the produced part features (i.e., thickness) in order to achieve homogeneous grain refinement [6]. In addition, cold rolling has also been used to control the parts width, and consequently, improve the surface finish of the final part geometry [25]. An example of a cold rolling variant, side rolling, is depicted in Figure 3, in which the material is strained in both longitudinal and normal directions.

An equally significant improvement of mechanical properties has also been made through the use of machine hammer peening [26,27] and laser shock peening [28]. Laser shock peening was performed laterally on 2319 post-machined aluminum parts and resulted in a decrease of average grain size by 22%, and in an increase of hardness [28]. This method, however, only had noticeable effects within the first millimeter of the wall and the induced plastic deformation was not enough to refine the complete wall thickness.

In summary, cold-work based techniques can significantly reduce residual stresses and distortion, improve microstructural homogeneity and mechanical properties, reduce porosity, minimize waviness, and increase final part geometry accuracy. However, most studies have been carried out on simple geometry parts and the use of this methodology may be therefore limited to specific designs. Heavy equipment is required when cold-working is to be applied, and its usage can cause an increase in lead times devaluing the main characteristic of WAAM: its high deposition rate.

#### 2.1.2. Active Inter-Layer Cooling and/or Heating

The high heat inputs and consequent low cooling rates typically result in coarse columnar grains and anisotropy. Moreover, in WAAM, by definition, the inter-layer temperature is the temperature of the previous deposited layer just before a new one is deposited, and is of major importance on the part’s final properties [29]. It determines the conditions of heat dissipation by conduction through the part, directly affecting the cooling rate and consequently, the microstructure and mechanical properties. Due to the difficulty in reducing heat accumulation, it becomes difficult to keep a low inter-layer temperature. This type of control is not suitable since a high inter-layer temperature may only be achieved with the use of double torch system. On the other hand, the time needed to cool down the part to a suitable temperature may lead to a total production time impracticable with extended idle times. Even though a high inter-layer temperature improves the wettability of the molten metal [30], when working with high temperatures the deposition becomes unstable and may even lead to the collapse of the wall. In a first approach, the inter-layer temperature can be controlled by imposing an interlayer idle time which can be optimized with the simulation of thermal behavior during part production using finite element models [29]. 

The utilization of process add-ons to control thermal cycles has already been tested, using compressed CO_2_ gas to impose a forced cooling. This promising development in WAAM of Ti-6Al-4V presents benefits including: better surface finishing with less oxidation, refined microstructure, improved mechanical properties, and enhanced manufacturing efficiency [31]. 

**Figure 4:**
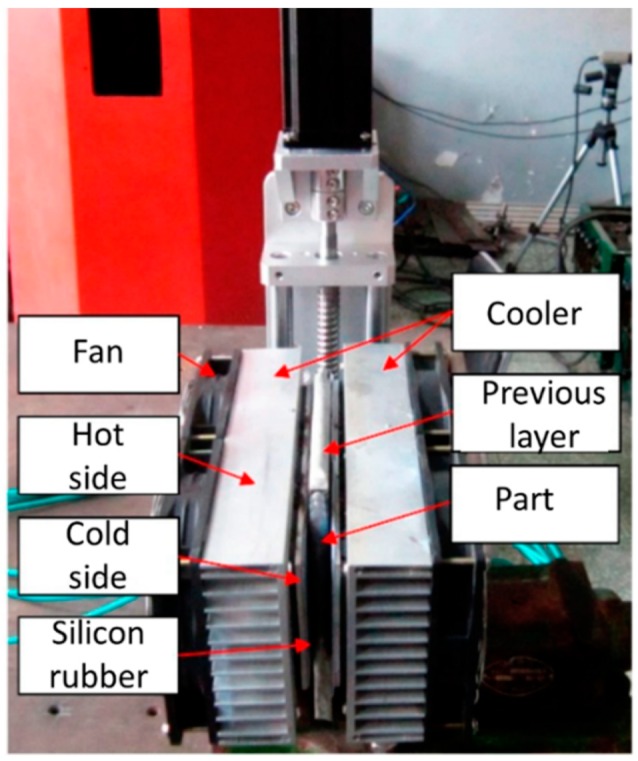
Representation of thermoelectric cooling setup (adapted from [32]).

Another alternative to control the thermal cycles in WAAM, is using thermoelectric cooling. Figure 4 depicts this approach. Heat sinks by conduction to the side walls, enabling similar heat dissipation conditions throughout the full deposition. Additionally, this technique allows the control of the bead geometry, decreasing the surface waviness by about 60% and the total fabrication time can decrease by nearly 60% as well [32], since a continuous heat dissipation condition is achieved without adjusting process parameters. 

**Figure 5:**
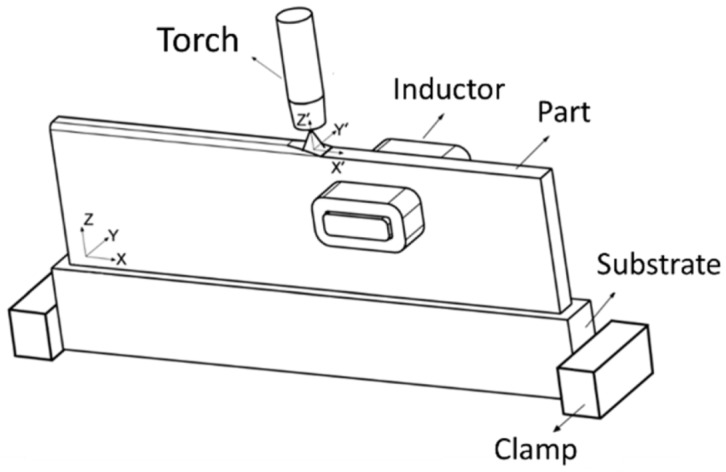
Schematic representation of the inductor heating system (adapted from [33]).

While the previous technique envisaged to cool down the temperature interlayers, for some materials and applications it may be of interest to have a quasi-isotropic material with minimum residual stresses without post processing heat treatment. In this case, an innovative method was developed to mitigate residual stresses consisting of an inductor with two symmetrical coils mounted on both sides of the as-built part, as schematically depicted in Figure 5 [33]. The inductor can perform both pre-heating and post-heating, depending on its positioning relative to the arc. In general, this technique reduces the residual stresses and distortion of as-built parts. Moreover, this system has the potential to overcome the inter-layer temperature issue, opening the possibility of maintaining a constant inter-layer temperature throughout the full deposition.

**Figure 6:**
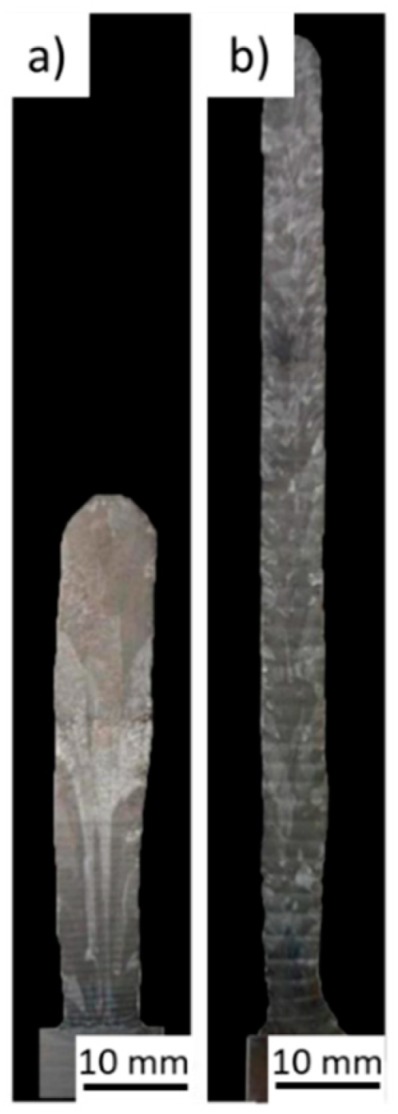
Macrostructure of samples produced: (**a**) with secondary heat source and (**b**) without (adapted from [34]).

Another process variant named hot-wire arc additive manufacturing (HWAAM) was developed and constitutes an efficient alternative to refine the typical columnar grains in Ti-based alloys processed by WAAM [34]. This variant consists on the use of another power source, which assists in melting the filler material, and reducing the amount of arc heat input in a GTAW-like application. The secondary power source has the positive pole connected to the filler wire while the negative one is connected to the substrate. Figure 6a,b depicts a comparison between samples built with and without this process variant. Besides a visible change in the wall geometry, due to different heat inputs, the size of columnar β-grains decreased in the sample built by HWAAM, resulting in a mixture of short columnar grains and equiaxed ones. This mixture promoted elongations of 12.6% and 12.8% in the longitudinal and transversal directions, respectively, in opposition to the 23% and 9.17% obtained with conventional WAAM, thus, confirming the appropriateness of this method to produce isotropic parts.

Localized heating/cooling mechanisms have shown promising results to reduce the interpass temperature that can cause parts to collapse and induce a heat treatment on previously deposited layers. Moreover, with only a constant interpass temperature it is possible to achieve more homogenous properties. However, this is a field where more research is necessary to manufacture parts with accurate pre-designed microstructures.

#### 2.1.3. Pre-Heating of the Substrate 

Pre-heating the substrate is one of the most efficient methods to mitigate residual stresses and cracking, since it reduces thermal gradients and homogenizes temperature distribution. Alberti et al. [35] compared depositions performed with and without pre-heating at a temperature of 300 °C in a PAW additive manufacturing process, and observed that pre-heating increased the wettability of each layer and enhanced the regularity of wall thickness, decreasing surface waviness. 

It is known that in WAAM, the width in the first layers is significantly less thick than the remaining layers due to a rapid cooling rate, which is caused by the large area of the substrate and its initial temperature. With pre-heating of the substrate, heat conduction decreases and heat losses are minimized, resulting in smaller temperature gradients. 

**Figure 7:**
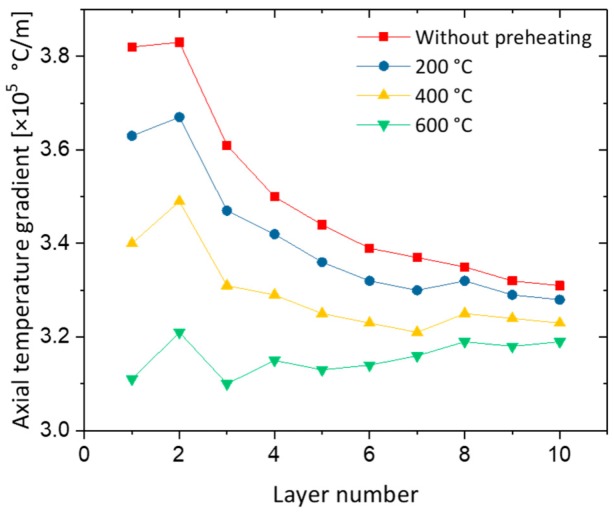
Temperature gradients variation from the first to the tenth layer (adapted from [36]).

Figure 7 presents the temperature gradients with the increase of layer height. The benefits of pre-heating the substrate on the first layers with a heat input of 570 J/mm is visible. The maximum temperature gradient in the first layer without pre-heating is 3.82 × 10^5^ °C/m and reduces to 3.63 × 10^5^, 3.40 × 10^5^, and 3.12 × 10^5^ °C/m with a pre-heating of 200, 400 and 600 °C, respectively. In addition, the temperature gradient decreases with the increase of deposited layers. Besides reducing the temperature gradients and achieving a smoother thermal cycle on the firsts layers, other benefits include reduced thermal stresses and cracking susceptibility [36].

#### 2.1.4. Substrate Release Mechanisms 

**Figure 8:**
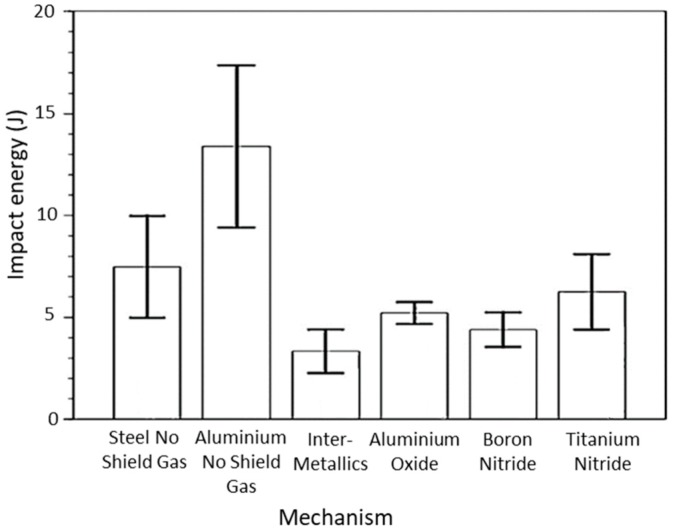
Impact energy test results of the samples built with different strategies and coatings (adapted from [38]).

Usually, the substrate has a composition similar to that of the material being deposited, supporting adhesion and stability. Even though novel design approaches consider the substrate as a part of the final component [37], Haselhuhn et al. [38] tested several ways to easily remove the produced parts from the substrates, reducing material waste. The authors evaluated the energy necessary, by the Charpy impact test, to remove aluminum parts by spray-coating aluminum oxide (18.50 µm thick), boron nitride (5.95 µm thick), and titanium nitride (6.25 µm thick) on an aluminum substrate. Each one was seen to assist in substrate parts removal, but there was not any statistical difference between the adhesion strength. Additionally, the effect of deposit on the first layers without shielding gas and with reduced voltage and current, in order to decrease weld penetration, was also investigated. The authors concluded that the arc instability caused by the lack of shielding gas reduced weld bead penetration. Finally, the effect of using dissimilar materials to promote the formation of brittle intermetallics between parts and the substrate was evaluated. According to the summarized results presented in Figure 8, the authors observed that the formation of intermetallics and the non-use of shielding gas in the first layers decreased the energy required to remove the parts produced from the substrate. This can be attributed to the mechanical properties of the intermetallics and the oxides formed by the inexistence of shielding gas, which tends to require lower amounts of energy to fracture, hence facilitating detachment between the substrate and the as-build part. In future works it is necessary to assess whether these mechanisms can be used for large-sized parts.

#### 2.1.5. Shielding Mechanism

Shielding gas is one of the most influent parameters, since it affects bead geometry, process stability, transfer mode, and bead appearance [39]. Besides its right selection, other methods have been used to improve WAAM parts quality. Xu et al. [40] studied the effect of oxides on the mechanical properties of maraging steel by varying shielding conditions. Results were obtained by making one deposition in the open atmosphere with pure argon, and the other in an argon-filled tent (chamber), in which the oxygen level was controlled below 300 ppm. Extra tent shielding substantially improved the surface waviness and deposition efficiency by 37% and 9%, respectively.

**Figure 9:**
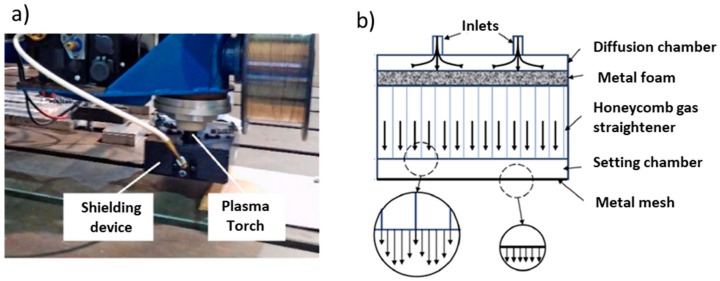
(**a**) Setup of the WAAM trailing shield device; (**b**) schematic representation of the shielding device (adapted from [39,41]).

Gas shielding flow was found to be of remarkable importance regarding wall appearance. In a preliminary study [39], the effect of gas shielding flow in WAAM was analyzed, since turbulence flow caused the shielding gases to mix with the surrounding air, resulting in poor shielding conditions and increased atmosphere contamination that can lead to oxidation. A new device (Figure 9) consisting of three distinct parts was developed to achieve laminar flow of the shielding gas. The first part is a diffusion chamber that uniformly distributes the inlet gas, the second is a honeycomb wall that straightens the flow and reduces its lateral velocity, and the last part, at the end of the chamber, is a layer of metal mesh used to further improve the uniformity of the flow. Overall, this new device decreased the level of contamination up to three orders of magnitude. 

As it can be observed, there are currently several impactful WAAM variants, all aiming to create parts with better mechanical properties and tolerances, and to reduce the need for post-processing techniques (machining and/or heat treatments). The abovementioned recent development shows the great activity and interest that WAAM is attracting, not only from academia, but also from the industry. Obviously, that focus cannot only be paid to the process variants in WAAM. The materials to be processed also play a critical role, and due to the vast possibilities of materials that can be used in WAAM, the same process variant may have different effects on the microstructure and/or mechanical properties of the parts. The following section is dedicated to the most recent developments regarding the different materials used in WAAM.

## 3. Materials

During parts fabrication, the deposited material undergoes various heating and cooling cycles that may result in different grain structures along their height. Grain structure control is of major importance since it determines the material mechanical properties. Typically, WAAM parts comprise large columnar grains, formed by epitaxial growth from the substrate aligned along the buildup direction normal to the solid/liquid interface, which has the maximum temperature gradient, thus eliminating the need for nucleation sites [42]. This type of growth results in anisotropic properties, which can be detrimental for multi-axial loading conditions. Equiaxed grains are desirable since they can reduce crack susceptibility while improving ductility, resulting in components with (near) isotropic properties. Thus, the use of add-ons that can perform in-process heat treatments, as well as post-WAAM heat treatments, is essential. Another practice to control the microstructure is the use of inoculants to refine the grain structure. A deep understanding of the mechanisms of nucleation with inoculants and grain growth, as well as the challenges for its use in additive manufacturing, is well described in [43].

Generally, any material available in the form of welding wire can be used for WAAM. The most used ones are steel, aluminum, titanium, and nickel-based alloys. Ti-based and Ni-based alloys are increasingly being studied due to their welcomed adoption by the aerospace industry. Thus, the desire to mature this process for its adoption into mass production of aerospace components comes from the ability to produce large parts with a low buy-to-fly (BTF) ratio. Other characteristics include, high specific strength, thermal and electrochemical compatibility with advanced composite materials, and the cost associated with applying subtractive methods onto these materials. 

One of WAAM advantages is the possibility to process a vast range of materials, therefore the present section reviews recent results, challenges, applications, and key details of the most commonly used metallic alloys for WAAM.

### 3.1. Titanium-Based Alloys

Titanium-based alloys are increasingly being study in WAAM, allowing for a reduction in the high costs associated with processing these materials. Ti-based alloys have high strength, toughness, good corrosion resistance, and can tolerate extreme temperatures without significant loss of mechanical properties, making them suitable for aerospace and biomedical applications [44]. Ti alloys represent around 15% of the total weight of the Boeing 787 [45], owing to its electrochemical compatibility with carbon fiber polymer composites.

Amongst the different additive manufacturing processes, WAAM allows for a better control of the microstructure of these polymorph alloys, since these materials are highly sensitive to the thermal history. Ti-6Al-4V is the most used Ti alloy and consequently the most studied in WAAM. Typically, it is constituted by two phases, a hexagonal close-packed structure (hcp), α, and a body-centered cubic (bcc), β. The different temperatures and cooling rates result in microstructure variations through the parts height. The most common microstructure comprises fine acicular or Widmanstätten colony and basket weave lamella-α morphologies [46]. Columnar β-grains from prior layers with grain boundary-α [47] are also prominent undesired features, causing premature failure in transverse loading solicitations [46,48]. This columnar structure is difficult to avoid since in low concentrations, aluminum and vanadium have a high solubility in titanium and do not partition ahead of the solidification front, becoming irrelevant as grain refiners [49]. Although the β grains transform to fine α during cooling below the β-transus temperature, primary β grains can still have a detrimental impact on mechanical properties [50]. 

Wang et al. [12] manipulated process variables to refine the poor primary β grains of Ti-6Al-4V in pulsed-GTAW, concluding that the peak/base current ratio and pulse frequency had no significant effect. However, equiaxed grains were achieved with a higher wire feed speed, since more nucleation sites were provided, blocking the columnar growth. 

**Figure 10:**
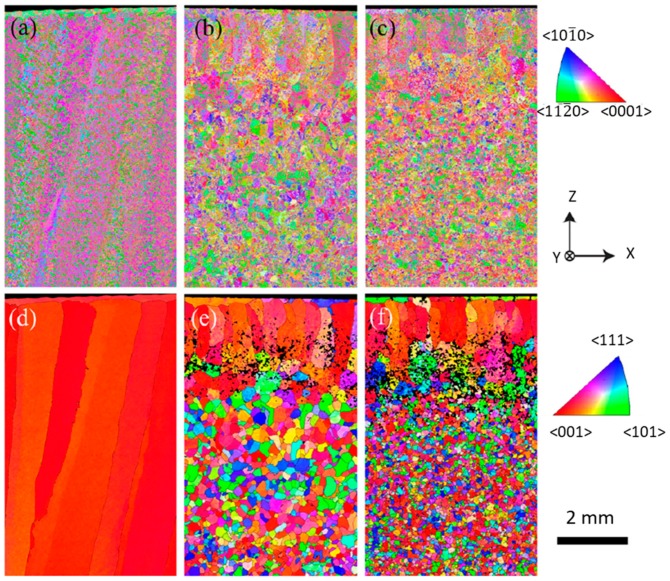
Electron backscatter diffraction (EBSD) maps of the α-phase (**a**–**c**) and reconstructed β-parent phase (**d**–**f**) of samples produced without rolling and with rolling loads of 50 kN and 75 kN (adapted from [51]).

From the previously described process variants, interpass rolling [51] showed to be a suitable process to mitigate the typical anisotropy of additive manufacturing parts including Ti-6Al-4V parts. Figure 10 depicts different electron backscatter diffraction (EBSD) orientation maps of both the α-phase and reconstructed β grain structures prior to transformation in an unrolled sample, rolled sample with 50 kN, and rolled sample with 75 kN. The β grains are represented by the color red. Reconstruction of prior β structures is often used to index this phase, due to its small scale and low volume fraction. The unrolled sample exhibited a β-phase with a strong preferential <001> crystallographic direction, and with columnar grains visible in both maps. When rolling was applied, new β orientations, associated with the deformation of α laths, were created and a strong columnar texture was mitigated. In a recent study, these new β orientations were found to arise from twinning with the residual β [52]. Another key effect observed was the refinement effect induced by the interpass rolling applied to the WAAM deposits: by increasing the applied load the grains become more refined, resulting in an improvement of the material microstructure.

The common anisotropy of additive manufacturing parts and potential presence of undesired phases can significantly reduce mechanical properties in both build up and transversal directions, urging the need to control the microstructure during fabrication. Potentially nucleant particles are being used in WAAM to refine the microstructure and enhance mechanical properties of Ti-based alloys. 

By adding boron traces (up to 0.13 wt.%), Bermingham et al. [53] demonstrated the effectiveness of inoculants in eliminating the anisotropic microstructures of Ti-6Al-4V. Boron had a significant impact on β-grain morphology and TiB needles were formed. These particles were found dispersed in the microstructure, allowed to nucleate α-grains, and produced isotropic α-microstructures. The boron modified alloy exhibited an increase of 40% in the failure strain, with the average failure stress maintaining around 850 MPa.

**Figure 11:**
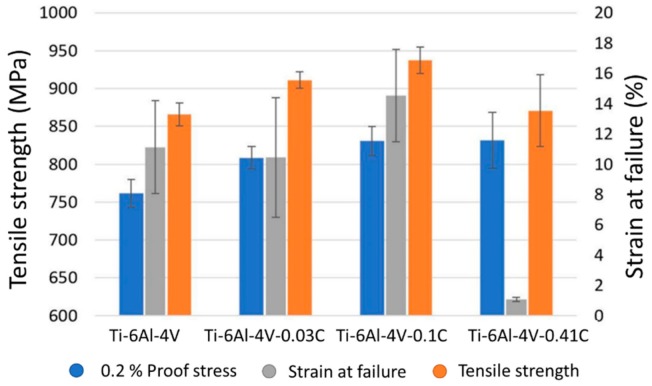
Effect of carbon additions on Ti-6Al-4V mechanical properties (adapted from [54]).

Mereddy et al. [54] added up to 0.41 wt.% of carbon in Ti-6Al-4V parts. The β-grain density increased while the α-lath length decreased. Carbon is an effective refiner in Ti alloys with hypereutectic compositions since it nucleates TiC particles. However, for hypoeutectic compositions, refinement is a result of the segregation of carbon solute, decreasing solidification temperature, and generating constitutional supercooling and growth restriction. The mechanical properties of as-built samples with and without carbon additions, with a small amount of carbon (0.03 wt.%), medium amount (0.1 wt.%), and excessive amount (0.41 wt.%), are illustrated in Figure 11. Sample built with 0.41 wt.% of carbon formed large carbides which significantly deteriorated the mechanical properties, while the part built with a medium amount of carbon had an increased strength and ductility of 9% and 30%, respectively.

Similarly, the same authors added silicon to a commercially pure titanium wire, promoting refinement of the grain size, particularly on the prior β grains [55]. Silicon, however, did not fully eliminate columnar grains. Instead they became narrower with a similar length to those of silicon-free samples. Overall, silicon promoted supercooling and growth restriction, but further refinement might only be possible with additional powerful refiners.

### 3.2. Nickel-Based Alloys

Nickel-based alloys are a class of materials mostly used in the aerospace and nuclear industries, for instance in transition ducts and gas turbines. These alloys are characterized by high strength at elevated temperatures, low thermal expansion, and excellent corrosion resistance. Their common austenitic matrix makes them suitable to operate within a wide range of temperatures. High costs, ability to adhere to cutting edges, and the presence of abrasive carbide particles makes Ni-based alloys difficult to machine, so WAAM becomes a viable technique to eliminate the material waste and consequently the overall costs associated with processing of this alloy. Upon solidification, Ni-based alloys may exhibit the following: solidification cracking [56], liquation cracking [57], ductility-dip cracking [58], and strain-age cracking [59]. Hence, special care must be taken during WAAM of these materials.

Typically, Ni-based alloys, such as Inconel 625 and Inconel 718, have high concentrations of alloying elements that can segregate during solidification in the interdendritic spaces. Moreover, their mechanical properties are highly governed by the Laves phase, and its morphology is dependent on thermal history which consequently affects the parts final properties. Inconel is a solid-solution-strengthened nickel-based superalloy, that with the addition of substitutional alloying elements, such as Cr and Mo, provides nucleation sites and the preservation of austenite once cooled. Other phases commonly found in Inconel that are used for strengthening effects, include γ′ phase [Ni_3_(Al, Ti, Nb)], γ″ (Ni_3_Nb, ordered bct D0_22_ structure), and blocky MC carbides. Nevertheless, the mechanical properties of Inconel alloys can decrease with the formation of undesirable phases, such as the δ-phase (Ni_3_Nb, orthorhombic) [60].

Inconel 625 parts manufactured by WAAM consist of vertically columnar dendrites in an austenitic phase (γ) matrix [61]. Solidification started with the L to γ reaction, and Nb and Mo precipitated in the interdendritic and grain boundaries where Laves phase will precipitate. With the increase of height during build up, the primary arm spacing varied from 13 µm, 23 µm, and 35 µm, from near the substrate, in the middle section, and in the top region, respectively. It obtained an average ultimate tensile strength of 722 ± 17 MPa and 684 ± 23 MPa in the travel and build directions, respectively. As for the elongation, 42.27 ± 2.4% and 40.13 ± 3.7%, respectively, were obtained. Fracture analysis revealed a ductile morphology with dimples. The shielding gas also played an important role on the final mechanical and geometrical properties of Inconel 625 parts. The ultimate tensile strength increased by 50 MPa when 97.5% Ar and 2.5% CO_2_ as shielding gas was used [62].

**Figure 12:**
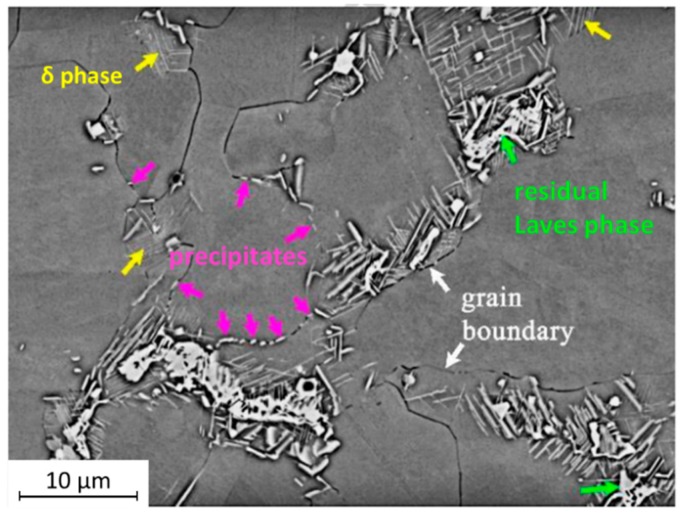
Precipitates found under SEM of the rolled with heat treatment sample (green: residual Laves phase, yellow: δ phase, purple: nanoscale precipitates along grain boundaries) (adapted from [63]).

Plasma arc-based WAAM was used to investigate the properties and microstructure of Inconel 718 superalloy in three conditions: as-built, with interpass rolling, and rolled with heat treatment in agreement with AMS-5662M [63]. The as-built samples exhibited the typical dendritic structure with Laves phase aligned with the build direction. The rolled samples showed refined grains near the boundaries of the produced walls with a recrystallized core in the central region. When solution plus aging heat treatments were applied, some of the Laves were dissolved and allowed to homogenize the distribution of the secondary phase particles. By X-ray diffraction, the δ phase was noticed after solution plus aging treatment of the rolled sample. However, its presence was not found in the wrought alloy. Laves phase, residual Laves phase, and δ phase precipitates of a rolled sample are depicted in Figure 12. Even tough rolling induced recrystallization, and columnar grains were still observed in some regions of the as-built samples, falling short to the wrought alloy. Consequently, the hardness was not homogenous along the wall thickness. Moreover, with the solution treatment, Nb did not completely diffuse and consequently Laves did not fully dissolve, suggesting the need for higher temperatures and/or longer periods for the heat treatment to be effective.

Table 1 presents the mechanical properties obtained for the as-built and rolled samples with and without heat treatment. With heat treatment, the average ultimate tensile strength was increased by around 284 MPa and 232 MPa more than the as-built samples, and by 266 MPa and 284 MPa more than the as-rolled samples in the longitudinal and transversal direction, respectively. Therefore, it was concluded that a significant improvement in the mechanical properties of Inconel WAAM parts can be achieved through proper heat treatment schedules and by paying special attention to the post-processing of these materials.

Xu et al. [64], evaluated the effect of oxides and different types of wires on the final properties of Inconel 718, since this alloy is known for its oxidation-assisted crack growth mechanism at high temperatures [65]. An oxide layer of Al_2_O_3_ and Cr_2_O_3_ with 0.5 μm thickness at the top layer was noticed, thus confirming that oxides do not accumulate during parts fabrication. By comparing different wires, differences of up to 50 MPa in the ultimate tensile strength were possible due to differences in chemical composition and uncertainties in TiN particles, which acted as nucleation sites.

Improvements in superalloys might only be achieved with thermomechanical processing. Even though Nb can diffuse, and consequently eliminate, Laves phases at interdendritic areas with heat treatment, the common large columnar grains will only coarse. Therefore, it is crucial to control Nb segregation and Laves formation in situ, since it depletes the matrix of useful alloying elements, or use of cold-work based techniques [66]. 

Another alloy of interest from the Ni-based group is Monel. These alloys, mainly composed of nickel and copper, are characterized by their high strength and excellent corrosion resistance at high temperatures, and are used in a wide range of aerospace applications. The ability to withstand corrosive environments makes them suitable for marine applications. Monel K500 and FM 60 were tested by means of cold metal transfer. The secondary dendrite arm spacing was smaller for the Monel K500 (4–9 μm) than for the FM 60 (6–12 μm), due to a higher precipitation of TiCN in Monel K500, which delayed dendrite growth. Energy dispersive X-ray spectroscopy (EDS) results showed segregation of Cu for both alloys, due to differences in the melting point of Ni and Cu, and the difficult diffusion of Cu in Ni. Precipitation of Ti-rich particles is essential to achieve superior mechanical properties and their density can increase with the use of inoculants. 

### 3.3. Steels

Steels are easily acquirable ferrous alloys widely used in automotive, ship, construction, and gas industries that, in combination with WAAM, can be used to manufacture parts with an overall low cost. However, some authors [67] claim that the production of these low-cost alloys by WAAM is only viable for large parts with complex geometries. Among steels, stainless steels have found applications in chemical plants and nuclear industries, where parts with high heat and corrosion resistance are required (e.g., pressure vessels). Austenitic stainless steel, such as SS 304 [68], SS 308LSi [69], and SS 316L [70,71] have been successfully used in WAAM, as well as martensitic stainless steel 420 [72]. 

Chen et al. [62] reported that 316 parts of stainless steel presented both austenite (γ), delta-ferrite (δ), and sigma (σ) phases with different morphologies at various positions as a result of the thermal cycles experienced during build up. After the fourth consecutive layer, the microstructure was composed of fine vermicular δ and σ phases within the γ matrix. With the increase of the parts’ height, the volume of σ increased, resulting in a decrease in strength and elongation. To avoid this phase, thermal cycles should be controlled to avoid long residence times between 600 and 900 °C. 

**Figure 13:**
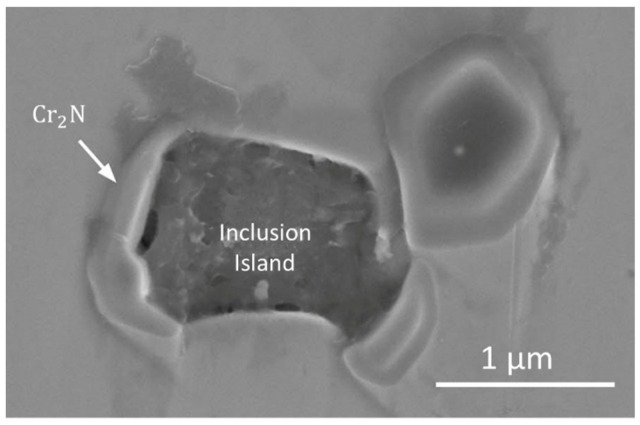
Cr_2_N precipitations around the inclusion island after heat treated at 1100 °C for 30 min (adapted from [73]).

The feasibility to produce high nitrogen Cr-Mn stainless steels was shown by Zhang et al. [73], where parts were mainly composed by dendritic δ-ferrite and columnar austenite, γ, with the existence of some CrN and Cr_2_N inclusion islands. The presence of oxygen in the shielding gas resulted in the formation of Mn-based oxides, which were found to be detrimental to the mechanical properties. Moreover, during post processing heat treatments the low solubility of nitrogen in δ-ferrite induced the formation of CrN and Cr_2_N in the nitrogen supersaturated regions. Due to the relatively high energy surfaces between the matrix and inclusions, Cr_2_N nucleated around Mn oxide inclusions, as observed in Figure 13. In conclusion, this material has shown to be deposited very effectively by WAAM with nearly isotropic characteristics due to a stable austenite matrix, while the presence of nitrogen had a work hardening effect.

The feasibility of WAAM to process steel tool production was demonstrated in H13 steel [74]. The results presented in this investigation suggest that the significant differences in properties along the part’s height are due to a different thermal history. The hardness varied between 300 and 360 HV and near-isotropic parts were obtained after annealing at 830 °C for 4 h. Average values of ultimate tensile strength and elongation were respectively, 1085 MPa and 10% for horizontal specimens, and 871 MPa and 7.8% for vertical ones. 

Maraging steels are a class of superior steel with high mechanical strength attributed to the presence of intermetallic compounds such as Ni_3_Mo, Ni_3_Ti, Fe_2_Mo, and Fe_7_Mo_6_. The aging temperature of maraging steel is relatively low (482 °C) and since this temperature is frequently exceeded during the process, overaging effects may occur. Using a plasma torch, Xu et al. [75] deposited maraging steel parts that revealed a martensitic matrix with fine residual austenite. With further aging, samples experienced an increase in the ultimate tensile strength of horizontal/vertical samples from 1118/1026 MPa to 1410/1345 MPa, while a decrease in elongation from 11.7/8.0% to 8.5/6.2% was observed. The authors reported the importance of avoiding undesired TiN inclusions in the feedstock material to obtain superior properties in the WAAM deposited parts.

Steels with a high carbon equivalent are more likely to experience cold cracking, a frequent problem due to rapid cooling, hydrogen entrapment in the heat affected zone, and residual stresses. Nevertheless, the procedures recognized to mitigate these problems in welding are intuitively used in WAAM, including pre- and post-heating, which is settled by re-heating or re-fusion of previously deposited material, which reduces the cooling rate, and subsequently the formation of brittle microstructures. Therefore, the intrinsic characteristics of WAAM are beneficial to avoid the aforementioned problems, though others may occur, such as overaging or precipitation of undesired phases as a result of the complex thermal cycles experienced during samples build up [76].

### 3.4. Aluminium Alloys

Welding of aluminum alloys (AA) has always been problematic, due to the formation of an aluminum oxide layer and solidification behavior. The use of WAAM in aluminum alloys is limited as porosity is of major concern. Such limitations have led to some investigations on the effect of heat treatments in WAAM Al parts. However, not every Al alloy is heat treatable. As it occurs in the welding of aluminum, during building of parts it is also preferred to use an alternate current (AC) [77] to remove the natural surface oxide film (alumina) which has a higher melting point. If not, melted remains are trapped inside the molten pool, resulting in pores and internal defects, which drastically decrease the parts mechanical properties. WAAM of Al alloys is very challenging due to the turbulent pool dynamics caused by the periodic inversing of polarity, which can result in decreases of the part accuracy. Other important properties regarding welding of the Al alloys include high thermal conductivity, high coefficient of thermal expansion, high solidification shrinkage, wide solidification temperature range, and high solubility of hydrogen [78]. 

Gu et al. [79] studied the influence of wire quality of final parts properties, highlighting that the pre-existence of undesired contaminants is a major driving force for hydrogen cracking. The main Al alloys used in the aerospace industry are 2xxx (Al-Cu) and 7xxx (Al-Zn) series alloys. However, they are highly susceptible to hot cracking if process parameters lead to high levels of thermal stress and solidification shrinkage. Nevertheless, Fixter et al. [80] successfully fabricated AA2024 parts without solidification cracking. The importance of these results arise from the fact that AA2024 is an unweldable alloy, and part production is enabled by the suitable selection of the Mg content in the feedstock wire.

**Figure 14:**
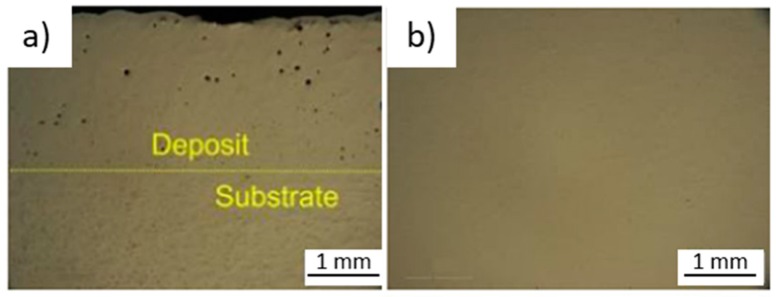
Porosity presence in samples manufacture with: (**a**) conventional cold metal transfer; (**b**) cold metal transfer-pulse advanced (adapted from [15]).

Cold metal transfer (CMT) is widely accepted as the most reliable variant to process Al alloys. However, CMT pulse advanced (CMT-PADV), developed by Fronius, was proven to entirely eliminate gas pores, due to an oxide cleaning effect [15]. Additionally, this variant is characterized by its low heat input and by the preservation of nucleation particles. Figure 14a,b depicts the macrostructure of aluminum with conventional cold metal transfer and with CMT-PADV, respectively, where the non-existence of pores with CMT-PADV is visible.

**Figure 15:**
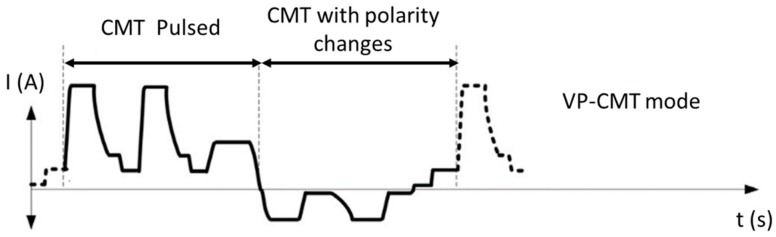
Current wave form of variable polarity cold metal transfer mode (adapted from [81]).

Zhang et al. [81] studied another similar power source mode named variable polarity cold metal transfer (VP-CMT). The typical waveform is presented in Figure 15. The pulsed arc mode results in an oscillation, that in association with alternating arc polarity changes, breaks the dendrite arms, providing heterogeneous nucleation sites. The typical columnar grains transformed into equiaxed and a grain refinement effect was observed. Despite isotopic grains confirmed by microscopy techniques, only a difference of 8% in ultimate tensile strength from horizontal and vertical samples was possible, due to the existence of interlayer pores.

The following Al-alloys deposited by WAAM have been reported in the literature: 5A06 [82,83] Al5Si [84], AA5183 [85], Al-Mg4.5Mn [20], Al-5Mg [86], Al-6Mg [81], and Al–6.3Cu [23]. In general, WAAM has its value in the production of Al parts, but the mechanical properties obtained are not always superior to the ones achieved from machining a billet, and the existence of add-ons, such as rolling, become important. The pressure applied, besides absorbing a significant amount of atomic hydrogen, also reduces porosity. Such has been experimentally verified for a load of 45 kN, where pores were eliminated to a level below the resolution of optical microscopy [14]. 

The effect of inter-layer rolling with different applied loads is clearly depicted in Table 2, which compiles the results of the number of pores, mean diameter, area percentage, and mean sphericity of WAAM deposited AA2319 and AA5087 alloys. Pores were completely eliminated when a 45 kN load was applied in between two consecutive deposited layers.

It is known that inter-layer rolling can provide nucleation sites that promote grain refinement and, depending on the inter-layer temperature, strain rate and applied load, different microstructures and properties can be achieved. The strengthening effects of inter-layer rolling for different WAAM deposited aluminum alloys are presented in Table 3. A linear improvement of the ultimate tensile strength and yield stress with an increase of rolling load in both cases, but with a consequential decrease in the elongation, is visible. Since inter-layer rolling is unable to uniformly deform beads, differences between longitudinal and transversal still subsist.

**Figure 16:**
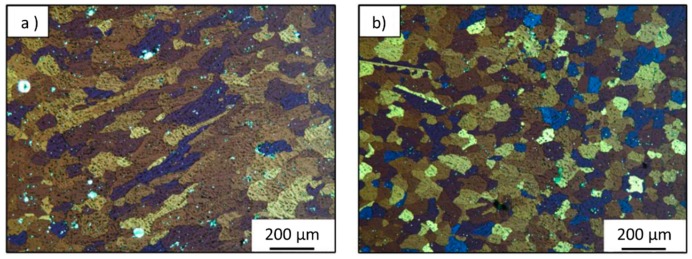
Microstructure of 5363 aluminum alloy: (**a**) as-built and (**b**) with Ti additions [86].

The microstructure and mechanical properties of 5363 (Al-5Mg) Al alloys were enhanced by adding titanium powder between layers [86]. Since Al_3_Ti and Al have similar face centered cubic (FCC) structures, formation of nucleation sites was improved. The addition of Ti resulted in the formation of fine equiaxed grains at the interlayer interface (Figure 16). The ultimate tensile strength and elongation respectively increased by 20.25 MPa and 3.13% in the horizontal direction and by 25.89 MPa and 6.97% in the vertical direction. This work highlights the viability to use inoculants to act as grain refiners during the production of Al WAAM parts in an attempt to obtain more isotropic and improved mechanical properties.

Sales et al. [87] aided the deposition of AA 5183 and AA 5356 with scandium which was responsible for the formation of Al_3_Sc intermetallic particles that acted as nucleation sites. The effect of adding zirconium and scandium as grain refiners was the same as using only scandium. The ultimate tensile strength and yield stress were increased by nearly 60 MPa in both horizontal and vertical directions.

### 3.5. Magnesium Alloys

**Figure 17:**
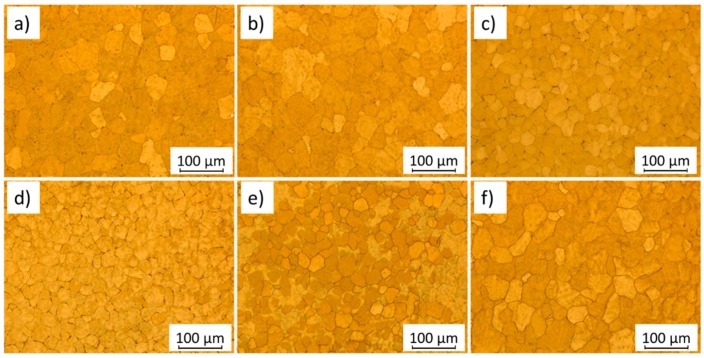
Microstructure of samples produced with a frequency of (**a**) 500 Hz, (**b**) 100 Hz, (**c**) 10 Hz, (**d**) 5 Hz, (**e**) 2 Hz, and (**f**) 1 Hz (adapted from [88]).

Magnesium alloys are increasingly being used as an alternative to aluminum to reduce the overall parts weight in the automotive and biomedical industries. Magnesium alloy advances throughout the years were hampered due to flammability risk, but with the increased interest in Mg-Al alloys, rare earth elements (zirconium, gadolinium, dysprosium, yttrium, neodymium and cerium), and other additional trace elements (Ca, Sr, Sb) were added to Mg suppressing ignition susceptibility. Magnesium is characterized by a hexagonal close-packed (hcp) structure and has few slip systems resulting in poor ductility. Owing to its structure, several defects can occur during forging or extrusion (i.e., edge cracking), therefore most magnesium products are processed via casting. Modified AZ31 and AZ61 are the most used Mg-alloys, however, only reports of the former processed by WAAM exists. As it occurs for aluminum, magnesium alloys also form an oxide refractory layer, but this can be removed easier than for aluminum. The necessity to refine magnesium structures was achieved by Guo [88] while presenting the feasibility to manufacture AZ31 WAAM parts. Such an achievement was obtained with GTAW technology by using six different pulse frequencies (1, 5, 10, 100, and 500 Hz), with the corresponding microstructures depicted, in Figure 17. Samples built with 5 and 10 Hz presented higher surface waviness, but the grain size was smaller and finer, measuring around 21 µm. The weld pool went through resonance with these frequencies, and as a result, the cooling rates decreased enhancing a finer structure. Sample built with 5 Hz exhibited an ultimate tensile strength of 258 MPa and an elongation of 25.6%, while sample built with 10 Hz exhibited an ultimate tensile strength of 263 MPa and an elongation of 23%. Overall, the samples produced indicated good plastic behavior well above the recommended value (234 MPa) stipulated by ASTM standard B91-12 [89].

Another already processed magnesium alloy with WAAM was the AZ91D [90], in which the authors highlight the remelting of previously deposited layers as the major problem with processing this materials. Furthermore, parts produced exhibited higher corrosion resistance than a cast magnesium sample, enhanced by the formation of Al_5_Mg_11_Zn_4_. Therefore, WAAM can be seen as a potential replacement of conventional casting processed in specific applications.

Rare elements are often added to Mg-based alloys. Since these are used for biomedical applications and are very susceptible to corrosion, ways to control and improve Mg alloys, rather than rare elements, is of major importance. 

### 3.6. Functional Graded Materials

**Figure 18:**
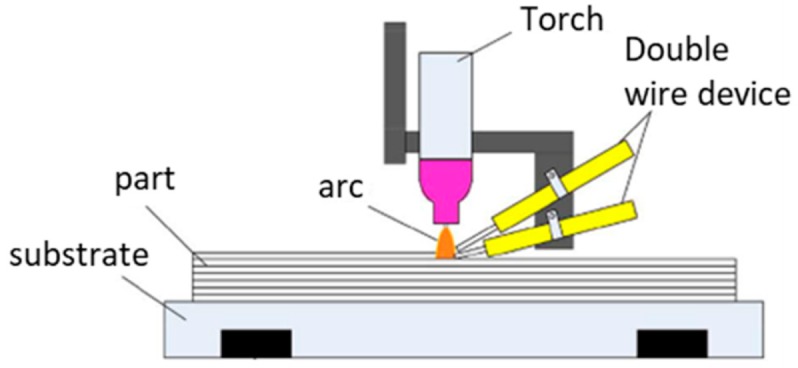
Schematic representation of double wire setup (adapted from [93]).

Besides producing bulked parts, WAAM is a suitable candidate to manufacture functional graded materials (FGM), which are an advanced class of heterogeneous materials which exhibit a controlled spatial variation of its properties (physical, mechanical, biochemical, among other) along at least one direction. Moreover, the manufacture of materials with site-specific properties is also possible [91,92]. Among the different additive manufacturing processes available today, production of FGMs is possible through binder jetting, directed energy deposition, material jetting, powder deposition, and sheet lamination. Wire and arc techniques offer unique advantages to manufacture FGM, due to their ability to vary the properties of the deposited material throughout deposition. The development of FGMs via WAAM can be obtained using the following two approaches: (i) by varying process parameters, such as wire feed speed or current; (ii) by feeding multiple wires, as schematically shown in Figure 18.

Somashekara et al. [94,95] used two separate wires with two independent power sources fitted into one torch in order to obtain flat pieces with a gradient of properties. Experimental studies allowed for a regression model to predict hardness as a function of torch speed and current of each wire with a maximum error of 6.5%.

A high purity annealed iron wire (99.5 at.%) and 1080 aluminum were combined under the electric arc of a tandem torch [96]. The content of aluminum was varied from 15 to 55 at.% every four layers by 5% increments. Such variation resulted in a compositional gradient with different intermetallics being formed in a variation of phases detected by X-ray diffraction. Near the substrate, specimens showed large columnar Fe_3_Al grains. With the increase of Al content phase changed to B2 structured FeAl and large columnar grains were eliminated, and when the content reached around 50 at.% FeAl_2_ was formed. With 36.1 at.% of Al content, an ultimate tensile strength of 315 MPa was feasible, but the mechanical properties of the specimens started to decrease rapidly for higher Al amounts. Specimens exhibited low ductility resulting in brittle transgranular lamellar fractures.

FGMs can potentially mitigate the issue of localized stress concentrations, as well as the manipulation of desirable properties and phases. However, when mixing dissimilar materials, some elements are likely to have different melting temperatures that when deposited may vaporize [97], requiring some attention. Additionally, the formation of brittle and/or undesired compounds greatly increases when fusion-based FGMs are being created. Therefore, it is critical to determine the range of process parameters that allow the production of complex shaped parts, exhibiting mechanical, chemical, or other graded property, while at the same time avoiding detrimental phases. To that sense, the use of a thermodynamic computational approaches [98] may be of great use to achieve the desired parts. 

### 3.7. Other Materials and Dissimilar Depositions

Apart from the already referred alloys, other metals have been studied, such as copper-aluminum (Cu-Al8Ni2Fe2) [70], a dissimilar deposition of stainless steel and Ni-based alloy [99], and lastly, NiAl bronze alloys (NAB) with potential for marine applications [8,100]. Aluminum-copper (ER2319) and aluminum-magnesium (ER5087) were fed into the same electric arc, and by adjusting the wire feed speed different chemical combinations were achieved (Al-3.6Cu-2.2Mg, Al-4Cu-1.8Mg, and Al-4.4Cu-1.5Mg) [93]. The phases in Al-3.6Cu-2.2Mg were mainly α-Al and S phase and with an increase of Cu and decrease of Mg content, θ phase gradually increased.

## 4. Deposition Strategy

The deposition strategy is critical in additive manufacturing processes based on fusion. In this section, recent studies and methodologies regarding the building strategy for WAAM parts are presented. 

Currently, there are several optimized softwares that slice 3D models and consequently create a G-code to be read by fusion deposition modeling printers. However, until now, there has not been a clarification about all WAAM constraints (e.g., residual stresses), in order to produce parts directly from CAD models. Kazanas et al. [101] proposed that the deposition of previous layers to occur in the form of pyramid to avoid the formation of humps in single inclined-walls. 

Another recurrent macrostructural problem occurs when every layer has the same start and end point. The excessive heat sink at the beginning of the deposition decreases weld penetration. In contrast, at the end of the layer, low heat dissipation due to high temperatures results in layer height drop. The inconsistent height will accumulate along the deposited layers, precluding the process. To overcome this issue, the current and travel speed should be higher in the beginning and reduced gradually at the end of the deposition [102]. Another way to mitigate this problem is through the use of a zig zag approach by switching in every layer the start and end points [103]. However, this last method can result in zones that have thermal accumulation promoting higher residual stress at the walls boundaries. An easy approach might be to consider these zones as sacrificial ones. 

Understanding bead geometry and its relationship with process parameters to improve quality of produced parts is of major importance during WAAM. Optimization of process parameters to obtain better surface quality is a rather a tedious work based on trial and error methods so, Geng et al. [83] unveiled two types of forming mechanisms named wetting and remelting, that are determined based on the remelting width in each deposition. Furthermore, the authors developed equations of beads cross sections based on process parameters, such as, wetting angle and radius of the spherical cap. The adequate material input and process parameters were calculated, so that the molten metal could spread vertically tangential to the spherical cap, improving surface waviness 

Nevertheless, WAAM parts are not uniquely built by single walls, and once understood bead geometry behavior, its then necessary to optimize torch tool path considering build orientation selection, build sequence, design constraints and if it will be necessary to conducted post machining. Mediocre planning may result in porosity, internal defects, lack of fusion between adjacent beads and high residual stresses, a topic that will be further described in the next section. A method named tangent overlapping model was proposed to approximate the bead cross-section by means of functions (parabola, cosine and arc) [104]. An optimal distance value between adjacent beads of 0.738 times the width of one bead was achieved in order to suppress the valleys beads. 

**Figure 19:**
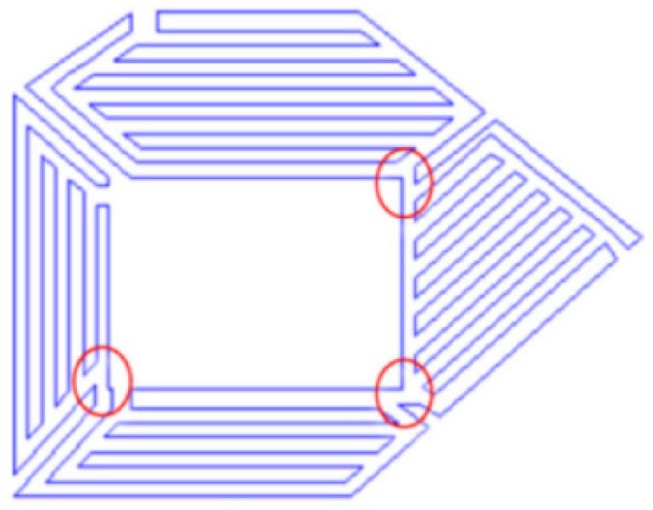
Convex polygonal method [105].

**Figure 20:**
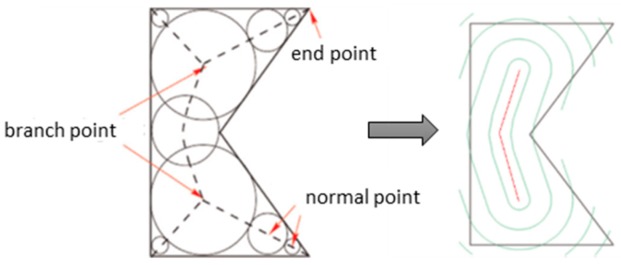
Schematic representation of the adaptive medial axis transformation planning method (adapted from [109]).

Regarding guidelines for the decomposition of CAD models, Ding et al. [105] presented a tool-path generation model that decomposed layers in polygons and each area was consequently filled. This model also automatically generated a final closed-looped tool-path by minimizing start/stops and crossovers of weld paths (Figure 19). A second approach on path planning modeling for WAAM was advanced by Ding et al. [106] through the improvement of earlier studies [107,108]. This method, named medial axis transformation, divides the geometry of a slice by producing a set of bisector segments. When more than two segments connect, those points become branch points and the paths will be generated by recursively offsetting contour-clockwise around the segment that connects two branch points as depicted in Figure 20.

**Figure 21:**
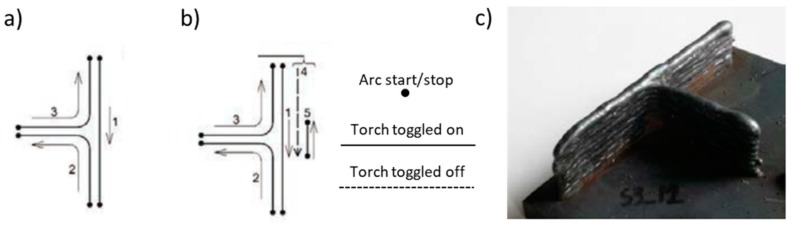
(**a**) First deposition strategy; (**b**) second deposition strategy; (**c**) final part made (adapted from [110]).

A path strategy regarding the manufacturing of 90° walls is presented in Figure 21 [110]. In order to assure a constant height for each layer, the strategy consisted in that after every fourth layers made with the first strategy (Figure 21a), the second deposition strategy (Figure 21b) was employed. Since one of the applications of WAAM is the fabrication of shell-part types, these successful developments are important, as they allow the production of smooth fillets of T-type structures. 

**Figure 22:**
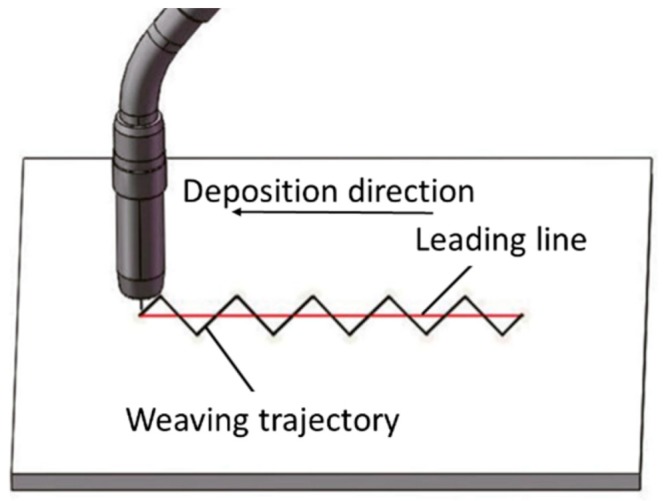
Weaving deposition strategy (adapted from [112]).

Other studies were performed to improve WAAM walls appearance, create T-type connections, and construct acute angles by adjusting the vertical distance and angle of the feed wire in a GTAW-based application. According to [82], an optimized angle of 10° with a distance of the melting wire tip to the molten pool surface of 3.8 mm guaranteed smooth layers. In a recent development, the influence of three different deposition building strategies (oscillation, parallel, and weaving) on surface waviness and porosity of maraging steels was studied [111]. Weaving deposition strategy, depicted in Figure 22, resulted in lower surface waviness and pores with reduced contact angle. In addition, Ma et al. [112] used weaving as a replacement for the multi-bead overlapping strategy to obtain better surface flatness, which required less post-machining, thus decreasing the associated production costs. 

In some additive manufacturing processes, there is a need to add material to support overhang volumes, that is later removed. Some research groups [37,113] studied the possibilities and advantages, and developed models in which the substrate was mounted on a 5-axis positioning system, which allowed the build direction selection during fabrication to change, thus eliminating the need for supporting material. With such a system, the correct selection of the substrate position becomes essential based on some criteria factors: mass of substrate waste, mass of the deposited material, the number of build operations, build complexity and symmetry. A detailed analysis of the correct position of the substrate may allow the BTF ratio to be reduced.

Since most of the software for metal deposition are limited and without public access, Yunyong et al. [114] took advantage of the free open source CuraEngine to introduce new settings to generate G-code to print metallic parts. These settings permit the torch to toggle on and off, path optimization to avoid crossovers in the same layers, the number of start/stops are minimized, and an option to pause the torch was also added for subtract cooling between layers. It is expected that these open-source software codes can be embraced by the additive manufacturing community to further expand its applications.

## 5. Residual Stresses

Residual stress challenges are of extreme importance within the WAAM process and are due to the complex thermal behavior and thermo-physical properties of the materials to be deposited [7]. Residual stresses are defined as stationary stresses at equilibrium in a portion of material after all external forces are removed [115]. Residual stresses are determined by their characteristic length: type I are macro-stresses varying over the dimensions of the component; type II are intergranular stresses; type III are formed at an atomic scale [116,117]. Despite the residual stresses that can be reduced by laser shock peening [28], in WAAM they can be as high as the yield strength of the material [118], negatively affecting the mechanical properties and leading to distortions and decreased tolerances. If these residual stresses exceed the local yield stress of the material, plastic deformation occurs, but if it exceeds the ultimate tensile strength then fracture is expected. These stresses are the result of the repetitive heating and cooling, which induce repetitive expansion and contraction of the material. For that reason, upon unclamping, the part balancing the internal residual stresses bends, and these can reach up to 500 MPa [22]. Considerable efforts are being made on strategies to mitigate this problem so that results can be further incorporated in path planning algorithms. Main findings include, that when building a layer, a pattern starting from the edges to the center cause less residual stresses on the substrate [119]. 

**Figure 23:**
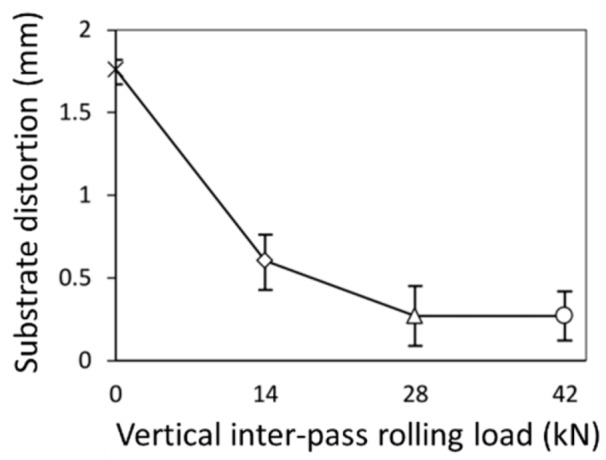
Substrate distortion with three different rolling loads (adapted from [19]).

**Figure 24:**
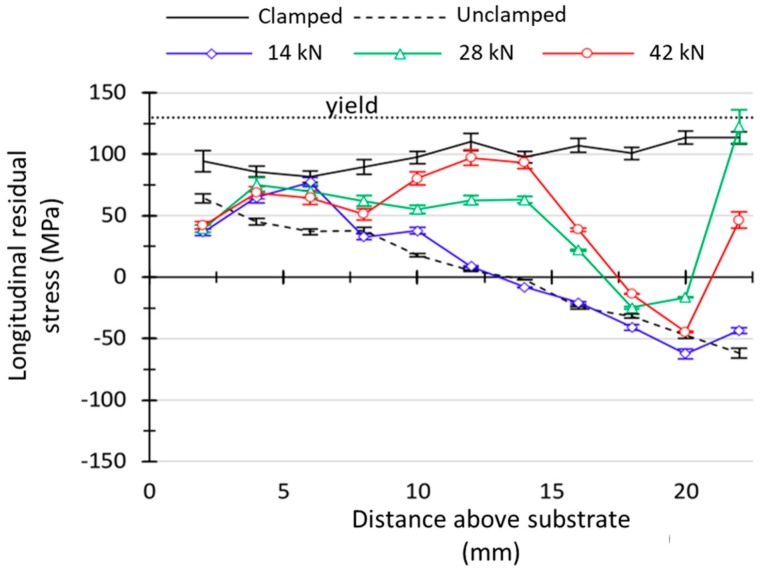
Longitudinal residual stresses of aluminum parts after rolling (adapted from [19]).

In others studies, several methods are employed in order to minimize the heat accumulation, and consequently residual stresses, based on the regulation of dwell time [120]; by pre-heating the substrate [36], since it reduces thermal gradients making the temperature distribution more homogeneous, it can increase the wettability of the first layers [35]; by mounting the substrate on a 5-axis system and building parts on both sides so that the residual stresses are balanced [37]. Other methods include the use of secondary heat sources to induce pre- or post-heating to obtain smoother temperature gradients [33]. Cold rolling is also used for the control of residual stresses in WAAM parts [19]. Figure 23 and Figure 24 depict substrate distortions and longitudinal residual stresses, respectively, with three different rolling loads. As-built samples experienced stresses near the yield strength in the longitudinal direction. A vertical rolling load of 28 kN was sufficient to mitigate distortion in the aluminum parts. Rolling allowed for a decrease in longitudinal stresses that become compressive near the top layers. However, rolling induced stresses in the transversal and normal directions that did not previously exist, indicating a three-dimensional stress state, which can significantly alter the mechanical properties of the parts.

Ultrasonic impact testing is a cold-work treatment that induces compressive stress on the top or on the side of the walls within a very limited depth, producing grain refinement. In WAAM it was seen to be effective in reducing residual stresses by nearly four times, compared to the as-built samples [121]. The energy of the arc was not seen to be enough to remelt all the recrystallized equiaxed grains of previous layers, forming a bamboo-like macro-structure.

## 6. Heat Treatments

During WAAM, excessive heat accumulation can prevent the accurate control of bead geometry [122], but also might lead to differences in mechanical properties and microstructure with the increase of samples’ height [123] becoming important to perform heat-treatments in order to achieve isotropic properties. These treatments might also be used to achieve the required mechanical properties, phases, relieve residual stresses, and remove internal defects.

In Section 2.1.2 in situ methods of heating and cooling WAAM parts were discussed, which can provide reliable and adequate heat treatments to achieve the required properties. However, these methods are still relatively recent and computational simulation developments are still necessary to predict phases and mechanical properties. Additionally, post-WAAM heat treatments can be used by means of hot isostatic pressing (HIP), solution treatment, annealing, aging, and other thermal treatments. 

**Figure 25:**
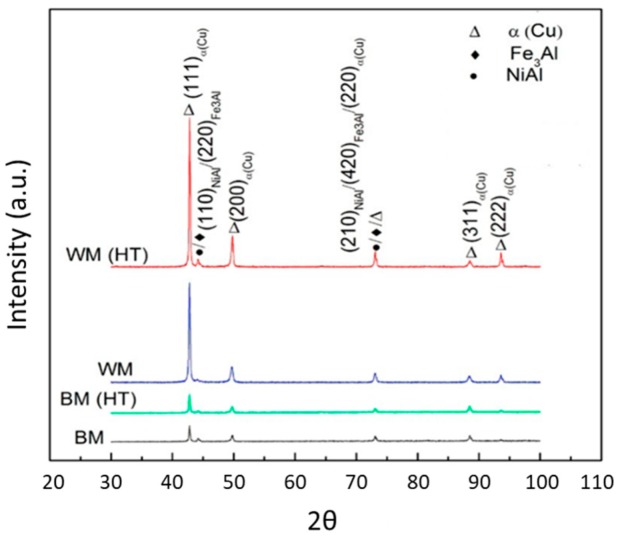
Phase constituents of as-cast base material (BM), as-deposited WAAM part (WM), heat-treated base material (BM HT), and heat-treated (WM HT) (adapted from [8]).

Gu et al. [14] studied the influence of post-deposition heat treatments on the porosity of 2319 and 5087 aluminum alloys, well-known for their porosity-related issues. After deposition, samples were kept for 90 min at 535 °C, followed by cold water quench. However, a combination of inter-pore coalescence (by Ostwald ripening) and hydrogen diffusion phenomena originated the growth of pores. Fully dense parts where only obtainable by combining inter-layer rolling and post-WAAM heat treatment. Other benefits from post-processing heat treatments were applied to NiAl Bronze WAAM components [8]. Parts fabrication annealing at 675 °C for six hours was applied, followed by air cooling, and resulted in the relaxation of the previously induced residual stresses in a fine homogenized microstructure composed of α phase (Cu) and intermetallics, such as NiAl and Fe_3_Al, as identified by X-ray diffraction (Figure 25). These constituents were not found in the base material, and their presence resulted in a significant increase in hardness from 181 HV for the base material to 210 HV after post-WAAM heat treatments.

**Figure 26:**
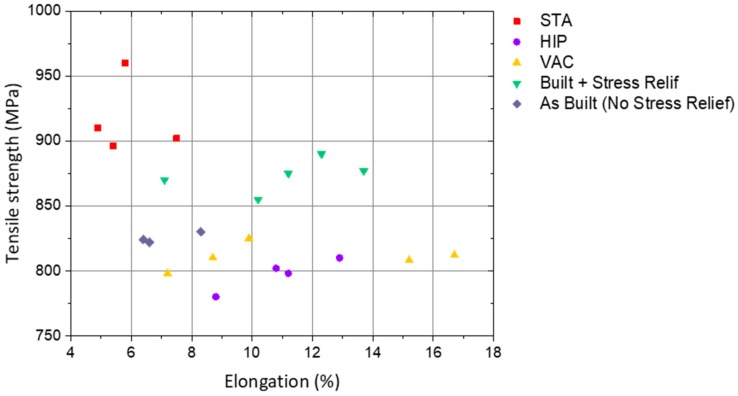
Mechanical properties of Ti-6Al-4V as-deposited and with post-process heat treatment (STA: solution treated plus annealing; HIP: hot isostatic pressing; VAC: vacuum annealing) (adapted from [124]).

Different post-processing heat treatments were used in WAAM parts of Ti-6Al-4V in [124]: stress relief (480 °C for 2 h); hot isostatic pressing (927 °C for 2 h at 1500 bar with a heating and cooling rate of 5 °C/min); vacuum annealing (927 °C for 2 h with a heating and cooling rate of 5 °C/min); and solution treated followed by annealing (967 °C for 1 h, water quenched then aged at 595 °C for 2 h and air cooled). The variation of mechanical properties was justified by changes in the microstructure, with the as-deposited sample exhibiting prior-β grains with fine Widmanstätten-α. The mechanical properties after each heat treatment are presented in Figure 26. The stress relief heat treatment resulted in a ductility increase of 30% and a microstructure similar to that of the as-deposited Ti-6Al-4V. Hot isostatic pressing effectively removed pores, and samples had a similar increase of ductility as vacuum annealed samples (around 40% of the as-built parts), explained by the continuously coarsening of the α-phase. Solution treated samples mainly comprised of refined α-phase grains, experienced an increase of 12% in strength, but in contrast the ductility was lowered by 30%. 

When mixing dissimilar alloys, it is likely to obtain intermetallic compounds [125,126,127]. Fe_3_Al is being increasingly studied with WAAM due to its unique properties [128]. In order to avoid some of the intermetallics and to reduce the anisotropy induced by the process, samples underwent homogenization (1000 °C for 7 h), homogenization plus transformation annealing in the FeAl region (850 °C for 24 h), and homogenization plus annealing in the FeAl region and a transformation treatment in the Fe_3_Al region (500 °C for 120 h), in accordance to the Fe-Al binary phase diagram. Homogenization on its own eliminated Al-rich precipitates and resulted in excessive grain coarsening. With further heat treatments in the FeAl-phase region these precipitates started to randomly appear. Finally, with only a third heat treatment Al-rich precipitates and new grain boundaries formed in an equiaxed shape with a size of 150 μm. In this study, excessive time of post-heat treatments was necessary to precipitate an adequate amount of Al-rich constituents that refined grain. Thus, from a manufacturer point of view, in situ methods to perform heat treatments should be developed and industrialized to save time and reduce costs.

**Figure 27:**
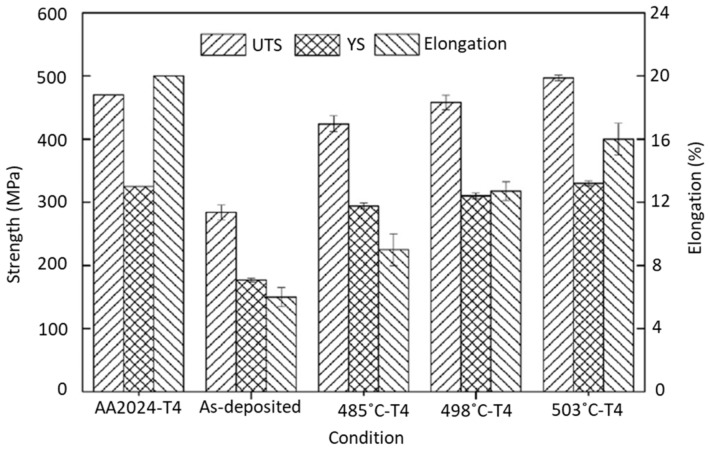
Tensile properties of 2024 aluminum samples [129].

The effect of heat treatments on Al parts fabricated by WAAM was also studied by Qi et al. [129], where solution treatments at different temperatures plus natural aging procedures (T4 condition) were applied to AA2024 parts. The effect of those heat treatments in the mechanical properties of the parts are evidenced Figure 27. All heat treatment conditions were seen to increase the mechanical properties compared to the as-deposit samples. From this study is it clear that, depending on which property (ultimate tensile strength, yield strength, or elongation) is to be maximized, proper selection of the heat treatment conditions is fundamental.

WAAM mostly uses alloys that can be heat treatable (e.g., high strength steels, titanium alloys, and nickel superalloys, for example) or non-heat treatable (e.g., austenitic SS and 4XXX aluminum alloys). To suppress columnar grains of non-heat treatable alloys, the development of cold working variants is of major importance. Regarding heat treatable alloys, superior properties that are difficult to obtain in as-build WAAM parts are expected. Further understanding of phase transformation kinetics that occur during the process is of major importance in order to avoid or reduce the need for post-process heat treatments. For this reason, the next section will focus on modeling and simulation applied to WAAM.

## 7. Modeling and Simulation for WAAM

WAAM parts’ microstructure and properties rely directly on process parameters, such as material, wire feed speed, heat input, and idle time between layers. The difficulties in optimizing a WAAM deposition and achieving the required characteristics, requires the execution of many experiments that can be highly costly and time consuming. Therefore, finite element analysis improvements are one of the primary goals toward a more massive adoption of WAAM. Typically, the process itself comprises melting of wire, metal transfer mode, mass transfer, gas absorption, convective flow of liquid metal, arc pressure, and solid-state phase transformations. The difficulty to model all phenomena comes from the diversity of materials and complex thermal behavior, since a portion of material is subjected to a vast number of thermal cycles of re-heating and cooling. Incorporating volumetric changes due to phase transformations can also be important [48]. Nowadays, most of the knowledge comes from trial and error experiments so if the process becomes fully modeled, accurate predictions of residual stress, distortion, and mechanical properties and microstructure can be achieved.

Chiumenti et al. [130] provided a detailed description of the formulation behind numerical simulations of WAAM processes while performing experimental validation. Their finite element model includes a heat transfer model governed by the laws of thermodynamics, Fourier’s law, Stefan-Boltzmann law, considered heat dissipation by conduction and convection, phase transformation phenomena based on Scheil’s equations, and the total amount of latent heat released/absorbed during phase changes. Additionally, a mechanical model which portrays a continuous damage model able to represent hot cracking singularities and porosities was included. The moving welding heat source was approximated by a double ellipsoidal power density distribution developed by Goldak et al. [131]. Montevecchi et al. [132] split the heat source into two power distribution contributions: one that characterizes the power delivered to the base material adapted from the double ellipsoid Goldak model and another constant of power distribution that should be developed to describe the energy transferred to the wire, since only 50% of the total energy was used to melt the wire [133]. 

These models are described as Equations (1) and (2), respectively:

(1) $${\dot{q}}_{b}=\frac{6\sqrt{3}{\dot{Q}}_{b}f_{f,r}}{\pi \sqrt{\pi }a_{f,r}\mathrm{bc}}\exp\left[-3\left(\frac{x^{2}}{a_{f,r}^{2}}+\frac{y^{2}}{b^{2}}+\frac{x^{2}}{c^{2}}\right)\right]$$

and

(2) $${\dot{q}}_{w}=\frac{{\dot{Q}}_{w}}{V_{\mathrm{el}}}$$

where: ${\dot{Q}}_{b}$ and ${\dot{Q}}_{w}$ are the analytic power value; coefficients a, b, and c are the semi-axis of ellipse dimensions, and $V_{\mathrm{el}}$ is the volume of heated elements.

Several finite element works have been conducted recently, such as evaluating the residual stress generated from clamping the substrate [134], or predicting the required idle time between layers to obtain a constant inter-layer temperature [29]. Zhao et al. [135] evaluated the stress distribution between single walls taking in consideration the deposition strategy: One wall was built always in the same direction, while the other had a deposition direction reverted in each layer. For both strategies, the stresses reduced with the height increase and it was experimentally concluded that layers in the same direction resulted in larger stresses than the part fabricated in the reverse direction. This suggests that deposition strategy is fundamental to mitigate residual stresses in WAAM parts.

Thermal models have been successfully developed to predict temperature gradients and distribution along WAAM walls, but there is a lack of models that can predict bead geometry. The difficulty comes with fully portraying heat transfer and fluid flow phenomena inside the molten pool. More recently, Bai et al. [136] succeeded in accurately predicting bead geometry by simulating heat transfer and fluid flow. Four different driving forces were considered: surface tension, Marangoni force, arc pressure, and arc shear stress. It was found out that metal liquid flow was upward inside and outward on the surface indicating that the Marangoni force dominates the fluid flow direction inside the molten pool. When depositing a high number of layers where side support is absent, the rear of the molten pool is driven by gravity and surface tension, which will determine the final bead geometry. 

**Figure 28:**
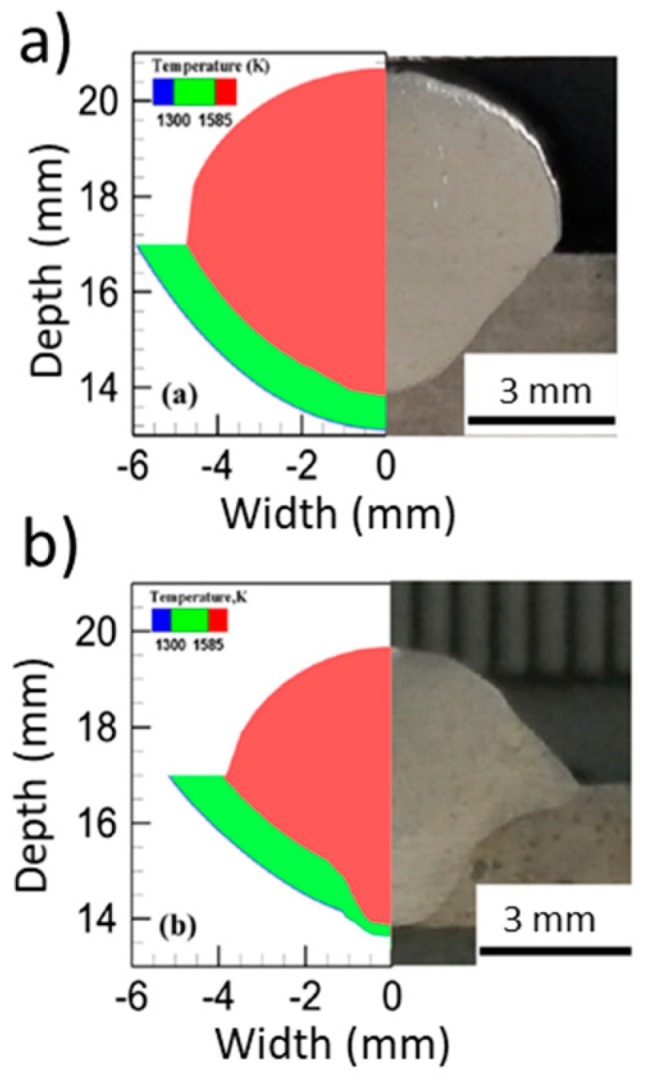
Comparison between predicted and experimental bead cross section for a travel speed of: (**a**) 5 mm/s and (**b**) 8.3 mm/s. (adapted from [137]).

In another approach, the buoyancy force and frictional dissipation in the mushy zone were included [137]. The geometry of the deposit material was simulated by means of the surface energy, potential energy in the gravitational field, arc pressure, and droplet impact force. Results have shown that by mixing hot and cold liquid inside the molten pool (convection inside the molten pool) reliable predictions of bead cross section were made. Figure 28a,b depicts the experimental results and numerical estimation of bead geometry for a traveling speed of 5 mm/s and 8.3 mm/s, respectively.

## 8. Integrated Machine

**Figure 29:**
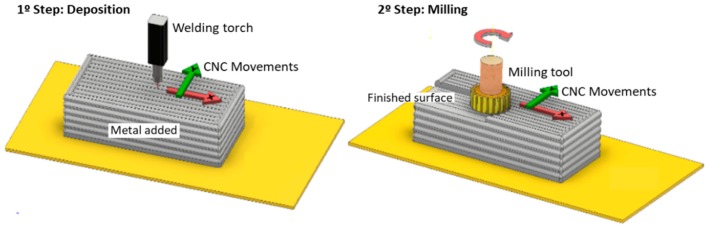
Steps in the hybrid process of metal additive manufacturing combining WAAM and milling (adapted from [138]).

Even though efforts have been made in optimizing tool path and process parameters, WAAM is sometimes referred as a near-net shape process due to its relatively poor surface finish. To answer the demands of short delivering times and complex parts, hybrid equipment which integrate additive and subtractive manufacturing technologies into one machine, arise as a promising solution to suppress WAAM’s major weakness: waviness. The typical process waviness can aid crack propagation, making post machining a necessity, especially in structural applications. A schematic illustration of this hybrid solution is presented in Figure 29. The present model could be improved by the existence of online measuring equipment, which can be used to regulate parts dimensions and amend dimensions with milling [112].

Chen et al. [139] highlighted the difficulty of machining parts with hollow cavities, pointing out the importance of sequentially depositing material and machining it, to avoid potential problems with machining after the entire model is produced. A hybrid manufacturing system was created, aided by a software developed at IIT Bombay, named arc hybrid layer manufacturing (ArcHLM) capable of generating paths for a welding torch and a milling tool [140].

In a cost modeling and sensitivity study, the economic benefits of WAAM with subsequent machining were compared to conventional machining from a solid block. The developed model considered the prices of tools, materials, substrate, machine, software, electricity, and every consumable for each manufacturing process. Several components were analyzed such as: a wing spar, an external landing gear assembly in titanium, and a pylon mount. In all studied cases WAAM represented costs savings compared to conventional machining as shown in Table 4 [141].

Mazak Corporation (Japan) unveiled a machine model VARIAXIS j-600AM featuring a standard WAAM head mounted on the machine headstock as well as an advanced 5-axis multi-surface subtractive capability to produce high-precision parts complete in single setups. Another advancement was made by Mutoh Industries that revealed the model Arc MA500-S1 which uses arc welding technologies. However, these machines are not versatile for research purposes, since no additional instrumentation or variation of the welding process is currently possible.

## 9. Defects and Non-Destructive Testing

As previously described, WAAM combined with a subtractive process is a viable solution to produce fully dense parts in an efficient way. The several process parameters of this process and complex material behavior leads to several challenges which can only be suppressed by a multi-disciplinary approach. Whilst there are no significant improvements on simulation software optimized for WAAM, integrating non-destructive testing and non-destructive evaluation sensors on equipment to evaluate parts as they are still being produced is of major importance. 

### 9.1. Defects

WAAM process is, fundamentally, very similar to welding so defects such as, hot cracking, cold cracking, porosity, delamination, and spatter are well documented for different alloys [142,143,144,145]. Defects in WAAM can originate by poor path planning, excessive heat input, and consequently residual stresses, gas contamination, and feedstock quality. Additionally, surface finish was seen to directly affect hydrogen crack susceptibility [146].

**Figure 30:**
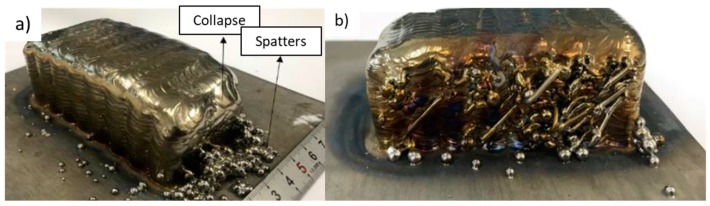
Image of WAAM macro defects: (**a**) side collapse; (**b**) unmelted wire (adapted from [147]).

**Figure 31:**
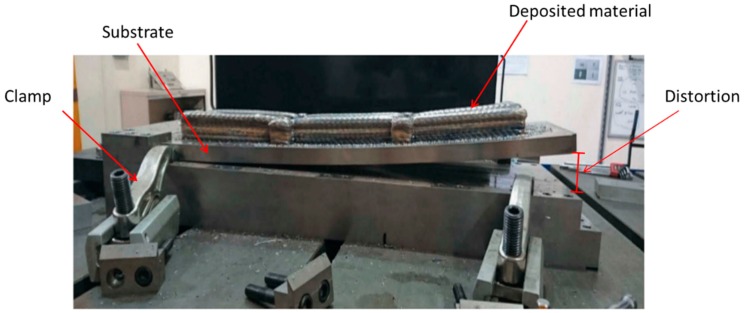
Distortion of a WAAM part (adapted from [148]).

Nevertheless, some predominant macro defects appear once layers start to be built in WAAM. These include side collapse, mainly caused by the excessive heat sink at the beginning of layers, in contrast to a low dissipation condition at the end of each layer (Figure 30a), that consequently results in unflatten top surfaces. Secondly, portions of unmelted wire can appear stuck to final parts, because of inconsistent wire stick-out on arc ignition. If the stick-out is too long the initial current will detach the wire without melting it (Figure 30b). Finally, large distortions upon unclamping as a result of the excessive heat input and consequently heat accumulation, result in a bending distortion of the component as depicted in Figure 31. 

The abovementioned defects can be mitigated with a correct choice of process parameters and process variants. Residual stresses that lead to distortions, and consequently to a loss of tolerance, can be relieved with post-processing heat treatments, right path planning, in situ cold/heating mechanisms, or even by the use of cold-work deformation-based techniques. Spatters are directly related with the selection of process parameters that will determine the transfer mode, which is easily overcome. Porosity is the most common defect for aluminum alloys and can be process-induced or due to poor wire batch quality. For Al-based alloys, rolling [23] and the welding mode CMT-pulse advanced [149] were seen to entirely remove pores. Side collapse and unmelted wire can be avoided by introducing sensors to assure a constant contact-tip-to-work distance, and a constant inter-layer temperature. Therefore, the following section describes the current status and difficulties on inspection and standardization of WAAM parts, covering in-situ monitoring and non-destructive testing as well as outline standardization and certification challenges.

### 9.2. In-Situ Monitoring

The main challenge of WAAM nowadays is developing appropriate inspection procedures. However, the increased capabilities to manufacture complex parts, decreases the ability for parts to be inspected. Therefore, using sensors to inspect parts as they are still being produced is of major importance. Everton et al. [150] reviewed in-situ monitoring and closed-loop control techniques for additive manufacturing, and some of them are yet to be exploited in WAAM.

It is well known that WAAM is very sensitive to variations of process parameters, so the implementation of an in-process monitoring device in a closed loop control system becomes important, since it would allow for rectification of parts as they are being still being built. Formation of internal defects and excessive residual stresses could be mitigated with such an approach. Traditionally, in fusion welding, monitoring is made through an evaluation of current, voltage, shielding gas flow rate, travel speed, and wire feed speed. In addition, during WAAM, sensors can be used to monitor the temperature at different regions [151], measure the size and geometry of beads [152,153], determine the weld pool characteristics, monitor the acoustic signal of deposition [154], detect electrical conductivity variations [155], and measure oxygen levels [156]. Normally, in fusion-based welding, the process parameters are held constant, but in WAAM, due to differences of thermal behavior throughout parts fabrication, geometric variations and mechanical properties are established and adjustments are necessary. Weld pool images contain abundant information that are directly related to parts quality. Therefore, the transient section of the weld pool was evaluated and monitored in [157], since defects such as cracks, delamination, and voids cause changes in heat flow and change the transient response. Thereby, on-line analysis of the arc spectrum was used to measure the weld pool characteristics by analyzing the abnormal bands according to the atomic emission spectrum, which contained necessary information about shielding gas flow rate and impurities that can influence fluidity of the weld pool and final parts quality. Spectrum analysis in combination with a CCD camera was able to detect material composition and confirm the presence of rust, oil stain, and shielding gas flow rate in the weld pool images [158]. An innovative single-neuron self-learning controller was integrated in WAAM equipment [159]. The developed controlling algorithm considered travel speed as the input control variable, and the layer width as the output to compensate layer height deviations. A non-linear Hammerstein model was used to establish a dynamic relationship between both parameters. The maximum deviations in layer width between the expected and detected layer width was 0.5 mm. 

**Figure 32:**
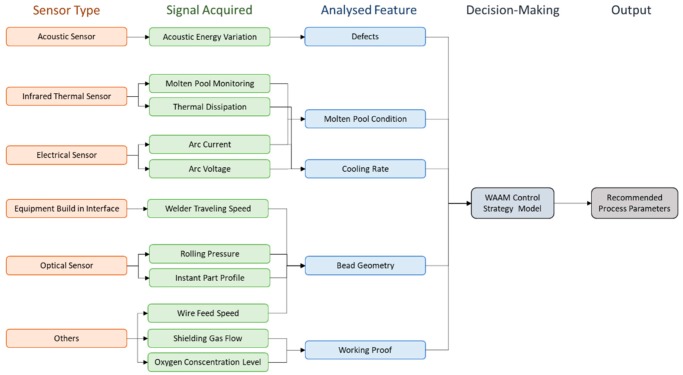
Schematic diagram of a full monitoring system for WAAM (adapted from [148]).

Xu et al. [148] reviewed process monitoring and control of WAAM parts and proposed a multi-sensor device to monitor each variant and output, as schematically shown in Figure 32. It considers an acoustic sensor for measuring arc pulsation and intensity, since when an irregularity occurs it will be reflected on the acoustic signal and on the signal of the current [154]. Infrared camera, thermocouples, or pyrometers would be used to monitor the molten pool and thermal cycles. In addition, it also contemplated a double profilometer and the use of add-ons, for instance, inter-layer rolling. Moreover, electrical conductivity measurements can be taken during deposition to identify the different phases that are being developed during solidification. In order to established WAAM consistency, a prior knowledge of the parts and build process is necessary to create an algorithm that will facilitate the industrial uptake of this process. 

### 9.3. Non-Destructive Testing

Non-destructive testing (NDT) comprises a set of a high valuable technique to assess parts integrity, and its development is necessary for the rapid industrialization of WAAM. The are several techniques (i.e., radiographic testing, tomography, ultrasonic testing, eddy currents, thermography and liquid penetrants) however, some of them have limited potential to be used in WAAM, mostly due to parts waviness as described by Lopez et al. [155]. These limitations include, for example, the need to inspect high temperature surfaces, which requires that the lift-off distance between the probe and the material significantly increases. The authors experimentally tested radiography and ultrasonic testing (UT) to detect surface defects in Al WAAM parts. Conventional ultrasonic testing is limited to off-line inspection, since couplants are susceptible to high temperatures, and require machined surfaces to be applied. Nevertheless, a novel solution was to place the probe on the bottom of the substrate, yet the signal corresponding to the interface of substrate and the part must be taken into consideration. Radiography could detect lack of fusions between layers and porosities, but to fully locate them it is necessary to analyze other sections. 

An ultrasonic array post-processing technique named total focusing method was seen to detect artificial holes with 3 mm, regardless of their location in part. Using conventional imaging techniques, defects near the side walls and top surface could not be detectable, and provided a non-realistic size and geometry of defects [160]. 

Currently, WAAM inline inspection conditions are extremely demanding for current state of the art NDT probes. The major issues include: high temperature of the last deposited layer; difficult to introduce a test medium to give a high signal-to-noise ratio; surface waviness and roughness; and small defects dimension (<1 mm). As such, the development of multiparametric non-destructive testing systems is mandatory to identify defects formation production and increase reliability in defect detection. 

## 10. Applications 

WAAM particularities makes it suitable for the creation of large-sized parts with medium complexity components, made with high-value materials. Therefore, this technology is viable to be used in industries, such as aerospace, automotive, defense, molds and dies, naval, and nuclear energy [70,161,162]. Topologically optimized structures have been increasingly being used by the aerospace and automotive industries as they reduce weight while maintaining the functionality of the part maximizing its performance. WAAM offers the potential to produce topologically optimized components, since through conventional technologies they become very expensive, with high material waste, and extensive lead times [163]. 

Currently, the aerospace industry focuses on the manufacture of complex-shaped components of titanium and nickel alloys, making WAAM a cost-effective method for their production, due to the difficulties associated with subtractive methods applied in these materials. Titanium represents 93% of the Lockheed SR-71 Blackbird’s structural weight and during its production approximately 90% of the forging weight had to be removed by machining [164]. Norsk Titanium delivered the first titanium additively manufactured component made via WAAM approved by the federal aviation administration, as it was installed on the Boeing 787 Dreamliner. Norsk Titanium’s technology allowed waste reduction, less energy consumption, and product costs reduction by up to 30% and 75% time saving, than forging with subsequent machining [165]. 

Ni-based alloys and stainless steels are widely used in the nuclear industry, where parts with high heat and corrosion resistance are required. WAAM is an appropriate candidate to replace some less requested portions of nickel parts to stainless steel, allowing the reduction of cost and weight of these components [99].

**Figure 33:**
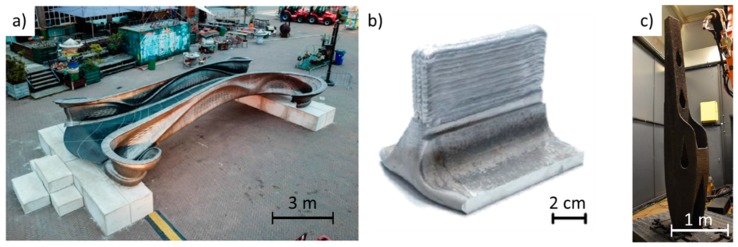
Various components made with WAAM: (**a**) MX3D bridge [168], (**b**) rib [166], and (**c**) excavator arm [169].

MX3D radically transformed the industry of construction by delivering the first additively manufactured metal bridge with a total weight of 4500 kg, 12.5 m in length, and 6.3 m wide (Figure 33a). Hirtler et al. [166] exploited one of WAAM’s possible applications, that is, manufacturing from a previous semi-finished component fabricated by a conventional metal forming process. A rib was first forged and then finished by increasing its height with WAAM (Figure 33b). Magnesium alloys have been used in WAAM [167], but its application for biomedical application still does not to meet the requirements in terms of corrosion-resistance and biocompatibility. Once these are ensured, those components can be used for human vertebra prototypes, hip stem implants, and treat bone fractures [168]. Another example included an excavator arm, as depicted in Figure 33c.

## 11. Summary and Future Outlook

The fabrication of complex shaped parts via WAAM is becoming well-established in both academia and the industry. Several process variants have been developed recently in order to optimize the microstructure and mechanical properties of the as-built parts. Additionally, most of the more relevant engineering alloys (titanium, aluminum, and steels, including stainless) are already used in WAAM with excellent results, proving the viability of this technique to produce custom-made large metallic parts. One aspect that is not yet well explored concerns the possibility of using WAAM for repair applications. This could greatly decrease costs associated with the need to completely renew a given structural part, since with WAAM technology it is possible to perform localized repairs.

Another key aspect that is not yet established is related to certification of WAAM parts. This step is crucial to further expand the range of applications of this technology and open the door for more demanding structural applications, where the advantages associated with WAAM can be of special interest. Concurrent to the need for certification procedures for WAAM parts, is the need to develop effective and integrated non-destructive testing systems capable of detecting defect formation during parts production. The need for the development of these in-situ monitoring methods lies in the fact that with such an approach any generated defect can be repaired right after its formation and not only at the end of production. Inspection only at the end of the parts production may lead to significant material waste and higher production times, since the location where the defect exists may be difficult to access, thus preventing their overhaul.

Some applications using WAAM-produced parts are already in the market and it is expected that industry plays a critical role in expanding the applications of WAAM parts. Combined with the development of certification procedures and effective non-destructive inline methods, it can be expected that WAAM will become one of the most used additive manufacturing technologies in the near future. 

## Figures and Tables

**Table 1:** Mechanical test results of Inconel 718 parts in the longitudinal (Long.) and transversal (Trans.) directions (adapted from [63]).

Process		YS 0.2% (MPa)		UTS (MPa)		Elongation (%)		Hardness (HV)
		Long.	Trans.	Long.	Trans.	Long.	Trans.	
As-built	unrolled	525 ± 7	506 ± 2	818 ± 13	756 ± 7	33.3 ± 2.5	27.9 ± 1.3	259 ± 8
	75 kN rolled	763 ± 8	687 ± 1	1082 ± 13	1072 ± 6	26.2 ± 2.2	26.6 ± 1.3	330 ± 19
Solution treated	unrolled	790 ± 9	791 ± 14	1102 ± 78	988 ± 6	14.7 ± 1.3	12.8 ± 1.2	417 ± 16
	75 kN rolled	1057 ± 19	1035 ± 20	1348 ± 10	1356 ± 10	15.1 ± 3.3	17.4 ± 1.1	443 ± 18

**Table 2:** Analysis results of pores for various stated WAAM 2319 and 5087 alloys [23].

Condition	As-Built		15 kN Inter-Layer Rolling		30 kN Inter-Layer Rolling		45 kN Inter-Layer Rolling	
Alloy	2319	5087	2319	5087	2319	5087	2319	5087
Number of pores (In a total area of 120 mm^2^)	614	454	192	336	5	11	Pores were completely eliminated	
Mean diameter (µm)	13.5	25.1	12.5	13	8.8	9.6		
Area percentage (%)	0.176	0.232	0.029	0.061	0.005	0.007		
Mean sphericity	0.74	0.74	0.67	0.63	0.37	0.42		

**Table 3:** Mechanical properties of different aluminum alloys with different build conditions in the longitudinal (Long.) and transversal (Trans.) directions.

Material	Variation	YS 0.2% (MPa)		UTS (MPa)		Elongation (%)		Ref.
		Long.	Trans.	Long.	Trans.	Long.	Trans.	
ER 2319	As-built	135	130	265	260	18.4	15.7	[23]
	rolled (15 kN)	146	140	270	265	15	14.8	
	rolled (30 kN)	185	170	290	280	13.2	11.8	
	rolled (45 kN)	250	245	322	310	8.6	7.3	
ER5087	As-built	142	-	291	-	22.4	-	[20]
	rolled (15 kN)	170	-	301	-	21.6	-	
	rolled (30 kN)	200	-	320	-	20.9	-	
	rolled (45 kN)	240	-	344	-	20.1	-	

**Table 4:** Costs of different parts per manufacturing process [141].

Designation	Process	BTF	Cost (€ × 1000)	Cost Reduction
Wing spar	Traditional	6.5	8.11	n/a
	WAAM	2.15	5.75	29%
External landing gear	Traditional	12	18.14	n/a
	WAAM	2.3	5.6	69%
Pylon mount	Traditional	5.1	2.8	n/a
	WAAM	1.5	2.68	7%
